# Effectiveness of Pre‐Transplant Dual GLP‐1 Receptor Agonist and SGLT2 Inhibitor Therapy on All‐Cause Mortality in Organ Transplantation Candidates with Obesity and Type 2 Diabetes: a Target‐Trial Emulation

**DOI:** 10.1002/advs.202518813

**Published:** 2025-12-12

**Authors:** Yu‐Nan Huang, Min‐Yu Tsou, Pin‐Hung Li, Jo‐Ching Chen, Yen‐Liang Liu, Gideon Meyerowitz‐Katz, Tsung‐Hsun Tsai, Pen‐Hua Su

**Affiliations:** ^1^ Division of Genetics and Endocrinology, Department of Pediatrics Chung Shan Medical University Hospital Taichung 402306 Taiwan; ^2^ School of Medicine Chung Shan Medical University Taichung 402306 Taiwan; ^3^ Cancer Biology and Precision Therapeutics Center China Medical University Taichung 406040 Taiwan; ^4^ Master Program for Biomedical Engineering China Medical University Taichung 406040 Taiwan; ^5^ School of Health and Society University of Wollongong Wollongong 2522 Australia; ^6^ Division of Urology, Department of Surgery Taichung Tzu Chi Hospital, Buddhist Tzu Chi Medical Foundation Taichung 427213 Taiwan

**Keywords:** GLP‐1 receptor agonists, organ transplantation, pre‐transplant optimization, SGLT2 inhibitors, type 2 diabetes

## Abstract

Evidence on pre‐transplant metabolic therapy remains limited. We evaluated whether concurrent glucagon‐like peptide‐1 receptor agonist (GLP‐1 RA) plus sodium–glucose cotransporter 2 inhibitor (SGLT2i) use before solid‐organ transplantation was associated with post‐transplant outcomes in adults with obesity and type 2 diabetes. A target trial is emulated using de‐identified electronic health records from TriNetX US. Dual therapy is compared with GLP‐1 RA, SGLT2i, and usual care using 1:1 propensity‐score matching. The primary outcome is all‐cause mortality at 12 months; kidney graft failure, rejection, complications, and infections are secondary. Sensitivity analyses included the global network, landmark, extensions at 24 and 36 months. Three matched cohorts are constructed, dual versus GLP‐1 RA (n = 4718 pairs), dual versus SGLT2i (n = 4282), and dual versus usual care (n = 3787). At 12 months, dual therapy is associated with lower mortality versus GLP‐1 RA (hazard ratio [HR] 0.69, 95% confidence interval [CI] 0.57–0.85), SGLT2i (0.59, 0.48–0.72), and usual care (0.52, 0.43–0.64). Infection endpoints are neutral or lower. Estimates are consistent across sensitivity analyses. In transplant candidates with obesity and type 2 diabetes, pre‐transplant GLP‐1 RA+SGLT2i use is associated with lower mortality than monotherapy or usual care. Prospective evaluation is warranted.

## Graphical abstract

This target trial emulation in solid organ transplant candidates with obesity and type 2 diabetes evaluates whether pre‐transplant dual therapy with GLP‐1 receptor agonists plus SGLT2 inhibitors is associated with post‐transplant mortality and kidney graft outcomes compared with monotherapy or usual care, using multinational electronic health records and extensive sensitivity analyses.

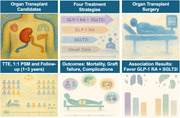

## 1 Introduction

Organ transplant candidates face a narrow window in which metabolic instability can erode the gains of modern surgery and immunosuppression.^[^ ^1^, ^2^, ^3^ ^]^ Obesity, diabetes, and the insulin‐resistant state that often follows advanced organ failure amplify peri‐operative infection, graft loss, and early mortality.^[^ ^4^, ^5^, ^6^ ^]^ Despite these risks, pre‐transplant metabolic care remains uneven and is seldom studied in prospective frameworks.^[^ ^7^ ^]^

Glucagon‐like peptide‐1 receptor agonists (GLP‐1 RAs) improve glycemic control, promote weight loss, and confer cardiovascular protection in large randomized trials.^[^ ^8^, ^9^ ^]^ Sodium–glucose cotransporter 2 inhibitors (SGLT2i) lower heart failure events and slow diabetic organ disease progression, with growing evidence of anti‐infective and anti‐inflammatory effects.^[^ ^3^, ^10^, ^11^, ^12^ ^]^ Their complementary mechanisms, one enhancing insulin secretion and satiety, the other promoting glycosuria and natriuresis, suggest a combined approach could offer additive benefits.^[^ ^13^, ^14^ ^]^ Recent studies have demonstrated the post‐transplant benefits of individual glucose‐lowering agents in kidney transplant recipients.^[^ ^7^, ^15^ ^]^ Large‐scale analysis of GLP‐1 RA or SGLT2i use showed associations with reduced all‐cause mortality and major adverse cardiovascular events in diabetic kidney transplant recipients.^[^ ^15^, ^16^ ^]^ Similarly, GLP‐1 RA therapy in type 2 diabetes and obese individuals without diabetes was associated with substantial mortality reduction, alongside cardiovascular protection including heart failure risk reduction.^[^ ^17^, ^18^, ^19^ ^]^ Yet transplant teams remain cautious. Concerns about volume depletion, gastrointestinal intolerance, and peri‐operative ketoacidosis have limited adoption, and no study has characterized the balance of benefit and harm when the two agents are used together before transplantation.^[^ ^20^, ^21^, ^22^ ^]^ This gap leaves clinicians without reliable estimates of infection risk, graft survival, or early mortality, the very outcomes that drive transplant success.^[^ ^7^, ^13^, ^23^ ^]^

We addressed this knowledge gap using target‐trial emulation with a multinational electronic health record network.^[^ ^24^, ^25^ ^]^ We compared recipients who received dual GLP‐1 RA and SGLT2i therapy in the two years before organ transplantation with propensity‐matched individuals treated with monotherapy or usual metabolic care.^[^ ^26^ ^]^ This approach allowed control of baseline imbalances while avoiding immortal‐time bias.^[^ ^27^ ^]^ Our primary aim was to determine whether dual therapy improves one‐year graft and patient survival without introducing excess safety events.^[^ ^28^ ^]^

## 2 Results

### 2.1 Baseline Characteristics After Propensity Score Matching

PSM achieved satisfactory balance across all comparisons, with standardized mean differences below 0.10 for measured covariates (**Table** **1**; Tables S6 and S7, Supporting Information).

**Table 1:** Baseline characteristics of organ transplant candidates with obesity and type 2 diabetes treated with dual GLP‐1 RA plus SGLT2i versus GLP‐1 RA monotherapy, before and after propensity score matching.

	Before Matching			After Matching		
	GLP‐1 RA + SGLT2i	GLP‐1 RA monotherapy	SMD	GLP‐1 RA + SGLT2i	GLP‐1 RA monotherapy	SMD
Characteristic	(n = 5230)	(n = 9218)		(n = 4718)	(n = 4718)	
**Age**						
Mean ± SD	59.9 ± 11.2	58.8 ± 11.7		60.0 ± 11.2	59.9 ± 11.1	
0 – 20 years	19 (0.4)	54 (0.6)	0.032	17 (0.4)	14 (0.3)	0.011
21 – 45 years	533 (10.2)	1149 (12.5)	0.072	484 (10.3)	467 (9.9)	0.012
46 – 65 years	2896 (55.4)	5084 (55.2)	0.004	2600 (55.1)	2613 (55.4)	0.006
≥ 66 years	1782 (34.1)	2931 (31.8)	0.048	1617 (34.3)	1624 (34.4)	0.003
**Sex (%)**						
Male	3269 (62.5)	4985 (54.1)	0.172	2884 (61.1)	2914 (61.8)	0.013
Female	1902 (36.4)	4084 (44.3)	0.162	1777 (37.7)	1757 (37.2)	0.009
**Race (%)**						
White	3000 (57.4)	5271 (57.2)	0.004	2710 (57.4)	2711 (57.5)	<0.001
Black or African American	1246 (23.8)	2087 (22.6)	0.028	1108 (23.5)	1090 (23.1)	0.009
**Socioeconomic determinants**						
Persons with potential health hazards related to socioeconomic and psychosocial circumstances	318 (6.1)	426 (4.6)	0.065	257 (5.4)	263 (5.6)	0.006
**Measures of healthcare utilization**						
Visit: inpatient encounter	2647 (50.6)	4388 (47.6)	0.060	2309 (48.9)	2312 (49.0)	0.001
Visit: emergency	1972 (37.7)	2989 (32.4)	0.111	1705 (36.1)	1729 (36.6)	0.011
Office or other outpatient services	3975 (76.0)	6680 (72.5)	0.081	3556 (75.4)	3558 (75.4)	<0.001
Emergency department services	2106 (40.3)	3165 (34.3)	0.123	1813 (38.4)	1827 (38.7)	0.006
**Comorbidities**						
Hypertension	4594 (87.8)	7554 (81.9)	0.165	4103 (87.0)	4077 (86.4)	0.016
Hyperlipidemia	3666 (70.1)	5477 (59.4)	0.225	3213 (68.1)	3193 (67.7)	0.009
CKD	3481 (66.6)	5974 (64.8)	0.037	3111 (65.9)	3122 (66.2)	0.005
IHD	2283 (43.7)	3150 (34.2)	0.195	1918 (40.7)	1921 (40.7)	0.001
Chronic lower respiratory diseases	1242 (23.7)	1906 (20.7)	0.074	1069 (22.7)	1055 (22.4)	0.007
Atrial fibrillation and flutter	992 (19.0)	1142 (12.4)	0.182	764 (16.2)	752 (15.9)	0.007
Mental and behavioral disorders due to psychoactive substance use	830 (15.9)	1241 (13.5)	0.068	720 (15.3)	708 (15.0)	0.007
Peripheral vascular diseases	536 (10.2)	904 (9.8)	0.015	478 (10.1)	466 (9.9)	0.008
Cerebral infarction	291 (5.6)	377 (4.1)	0.069	236 (5.0)	240 (5.1)	0.004
**Medications**						
Beta‐blocking agents	3580 (68.5)	5941 (64.5)	0.085	3150 (66.8)	3169 (67.2)	0.009
ACE inhibitors	1401 (26.8)	2016 (21.9)	0.115	1232 (26.1)	1251 (26.5)	0.009
ARBs	2013 (38.5)	2327 (25.2)	0.287	1607 (34.1)	1591 (33.7)	0.007
ARBs, other combinations	341 (6.5)	116 (1.3)	0.275	123 (2.6)	115 (2.4)	0.011
Thiazides, plain	740 (14.1)	1169 (12.7)	0.043	627 (13.3)	653 (13.8)	0.016
Aldosterone antagonists and other potassium‐sparing agents	1059 (20.2)	1075 (11.7)	0.236	752 (15.9)	778 (16.5)	0.015
Antiarrhythmics, class I and III	3449 (65.9)	5667 (61.5)	0.093	3024 (64.1)	3030 (64.2)	0.003
HMG‐CoA reductase inhibitors	4077 (78.0)	6023 (65.3)	0.283	3599 (76.3)	3613 (76.6)	0.007
Biguanides	2069 (39.6)	2458 (26.7)	0.277	1749 (37.1)	1742 (36.9)	0.003
Sulfonylureas	951 (18.2)	1347 (14.6)	0.097	833 (17.7)	862 (18.3)	0.016
DPP‐4 inhibitors	830 (15.9)	1195 (13.0)	0.083	737 (15.6)	748 (15.9)	0.006
Thiazolidinediones	231 (4.4)	295 (3.2)	0.064	203 (4.3)	198 (4.2)	0.005
Immunosuppressants	3479 (66.5)	5765 (62.5)	0.083	3140 (66.6)	3110 (65.9)	0.013
Sulfonamides	2675 (51.1)	4370 (47.4)	0.075	2313 (49.0)	2356 (49.9)	0.018
Antineoplastic agents	1057 (20.2)	1494 (16.2)	0.104	906 (19.2)	912 (19.3)	0.003
Insulin	4190 (80.1)	6522 (70.8)	0.219	3709 (78.6)	3700 (78.4)	0.005
Metformin	2069 (39.6)	2458 (26.7)	0.277	1749 (37.1)	1742 (36.9)	0.003
Glipizide	631 (12.1)	910 (9.9)	0.070	554 (11.7)	570 (12.1)	0.010
Aspirin	2516 (48.1)	3882 (42.1)	0.121	2157 (45.7)	2167 (45.9)	0.004
COVID‐19 vaccine	1053 (20.1)	1492 (16.2)	0.103	892 (18.9)	881 (18.7)	0.006
**Laboratory**						
**BMI**						
Mean ± SD, kg m^−2^	32.4 ± 6.0	33.5 ± 6.3		32.5 ± 6.0	33.2 ± 6.3	
≥ 30 kg m^−2^	3689 (70.5)	6663 (72.3)	0.039	3317 (70.3)	3316 (70.3)	<0.001
**ALT**						
Mean ± SD, U L^−1^	29.4 ± 87.4	27.9 ± 39.8		28.7 ± 83.4	28.0 ± 38.6	
≥ 30 U L^−1^	2705 (51.7)	4289 (46.5)	0.104	2390 (50.7)	2405 (51.0)	0.006
**Creatinine**						
Mean ± SD, mg dL^−1^	1.4 ± 1.6	2.0 ± 4.0		1.5 ± 1.7	1.8 ± 2.5	
≥ 1.5 mg dL^−^	2586 (49.4)	4786 (51.9)	0.050	2331 (49.4)	2340 (49.6)	0.004
**HbA1C**						
Mean ± SD, %	7.6 ± 1.7	7.4 ± 1.8		7.6 ± 1.7	7.6 ± 1.8	
≥ 7%	3076 (58.8)	4449 (48.3)	0.213	2736 (58.0)	2754 (58.4)	0.008
**TG**						
Mean ± SD, mg dL^−1^	181.8 ± 148.3	177.4 ± 124.0		181.7 ± 146.6	180.5 ± 124.7	
≥ 200 u L^−1^	1652 (31.6)	2479 (26.9)	0.103	1471 (31.2)	1473 (31.2)	<0.001
**LDL‐C**						
Mean ± SD, mg dL^−1^	76.7 ± 33.9	82.8 ± 37.6		77.4 ± 34.1	79.4 ± 35.0	
≥ 190 mg dL^−1^	76 (1.5)	177 (1.9)	0.036	68 (1.4)	66 (1.4)	0.004
**CRP**						
Mean ± SD, mg L^−1^	36.7 ± 61.9	37.3 ± 62.6		36.8 ± 62.9	37.1 ± 64.4	
≥ 3 mg L^−1^	823 (15.7)	1368 (14.8)	0.025	724 (15.3)	713 (15.1)	0.006
**BNP**						
Mean ± SD, pg mL^−1^	428.6 ± 1355.8	442.4 ± 1323.8		422.3 ± 1458.1	423.6 ± 1271.0	
≥ 100 pg mL^−1^	649 (12.4)	833 (9.0)	0.109	513 (10.9)	496 (10.5)	0.012

GLP‐1 RA, glucagon‐like peptide‐1 receptor agonist; SGLT2, sodium‐glucose cotransporter 2; SMD, standardized mean difference; IHD, ischemia heart disease; CKD, chronic kidney disease; HMG‐CoA, 3‐hydroxy‐3‐methylglutaryl coenzyme A; ACE inhibitors, angiotensin‐converting enzyme inhibitors; ARBs, angiotensin II receptor blockers; DPP‐4 inhibitors, dipeptidyl peptidase‐4 inhibitors; COVID‐19, coronavirus disease 2019; BMI, body mass index; HbA1c, glycated hemoglobin A1c; LDL‐C, low‐density lipoprotein cholesterol; ALT, alanine aminotransferase; TG, triglycerides; CRP, C‐reactive protein; BNP, B‐type natriuretic peptide. Percentages are rounded to one decimal place. SMD is rounded to three decimal places.

**Figure 1:**
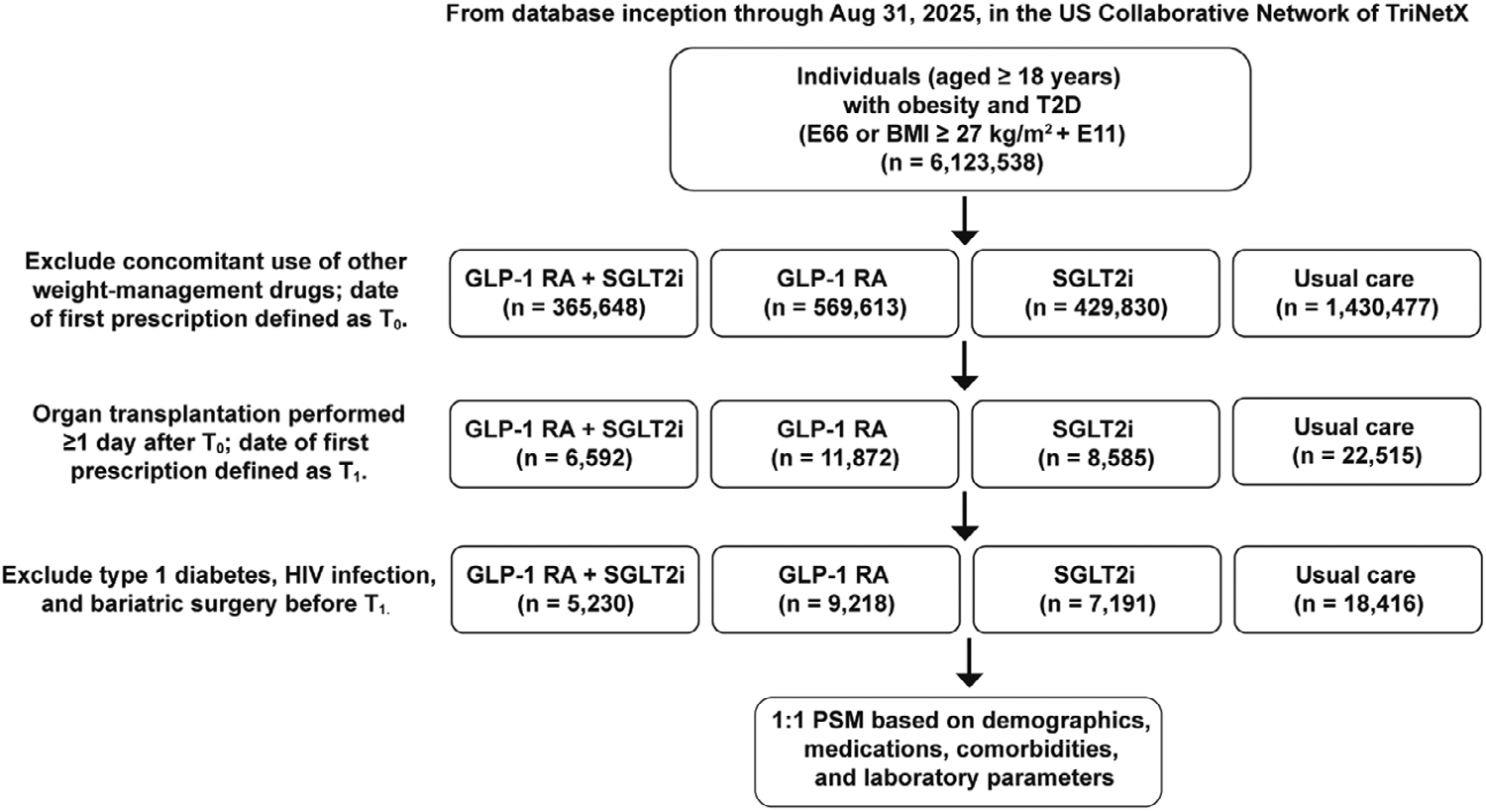
Study cohort selection and matching. From database inception through August 31, 2025, in the TriNetX US Collaborative Network, we identified adults (≥18 years) with obesity and type 2 diabetes (E66 or BMI ≥27 kg m^−^^2^ plus E11; n = 6123538). After excluding concomitant use of other weight‐management drugs, incident exposure at the index date (T_0_) classified patients into four groups: dual GLP‐1 RA + SGLT2i (n = 365648), GLP‐1 RA (n = 569613), SGLT2i (n = 429830), and usual care (metformin or sulfonylurea; n = 1430477). Among those who subsequently underwent organ transplantation ≥1 day after T_0_ (transplant date defined as T_1_), the cohorts comprised 6592, 11872, 8585, and 22515 individuals, respectively. After excluding type 1 diabetes, HIV infection, and bariatric surgery before T_1_, the eligible populations were 5230 (dual therapy), 9218 (GLP‐1 RA), 7191 (SGLT2i), and 18416 (usual care). These cohorts then underwent 1:1 propensity score matching based on demographics, medications, comorbidities, and laboratory parameters to construct the three pairwise comparison sets for the target‐trial emulation of pre‐transplant dual metabolic therapy. These cohorts underwent 1:1 propensity score matching based on demographics, medications, comorbidities, and laboratory parameters using nearest‐neighbor algorithms. The matching process generated three primary comparison cohorts with balanced baseline characteristics for robust statistical analyses of transplant outcomes. Final matched pairs included: dual therapy versus GLP‐1 RA monotherapy (4718 pairs), dual therapy versus SGLT2i monotherapy (4282 pairs), and dual therapy versus usual care (3787 pairs). BMI, body mass index; E11, type 2 diabetes mellitus ICD‐10 code; E66, obesity ICD‐10 code; GLP‐1 RA, glucagon‐like peptide‐1 receptor agonist; HIV, human immunodeficiency virus; PSM, propensity score matching; SGLT2i, sodium‐glucose cotransporter‐2 inhibitor; T2D, type 2 diabetes.

Across all three pairwise comparisons, large pre‐matching imbalances were observed in demographics, comorbidities, medications, and laboratory values—most notably versus SGLT2i (e.g., age ≥66 years, SMD 0.19, BMI ≥30 kg m^−^^2^, 0.21, HbA1c ≥7%, 0.33, insulin use, 0.34) and versus usual care (CKD 0.42, HbA1c ≥7% 0.44, sulfonylureas 0.49, immunosuppressants 0.38, COVID‐19 vaccine 0.41). After 1:1 nearest‐neighbor propensity score matching, we obtained 4718 pairs for dual therapy versus GLP‐1 RA, 4282 pairs for dual therapy versus SGLT2i, and 3787 pairs for dual therapy versus usual care, with covariate balance achieved across all variables (all SMD ≤ 0.05). Previously imbalanced features–‐including insulin exposure, HbA1c ≥7%, BMI ≥30 kg m^−^^2^, CKD, and use of sulfonylureas or immunosuppressants–‐were reduced to negligible differences (e.g., insulin, SMD ≤ 0.01; HbA1c ≥7%, ≤ 0.02; BMI ≥30, ≤ 0.01). These post‐matching cohorts provide comparable baselines for outcome analyses (**Figure** **1**).

### 2.2 Intention‐to‐Treat (ITT) Analysis for 1, 2, 3 Years Follow‐Up

**Figure 2:**
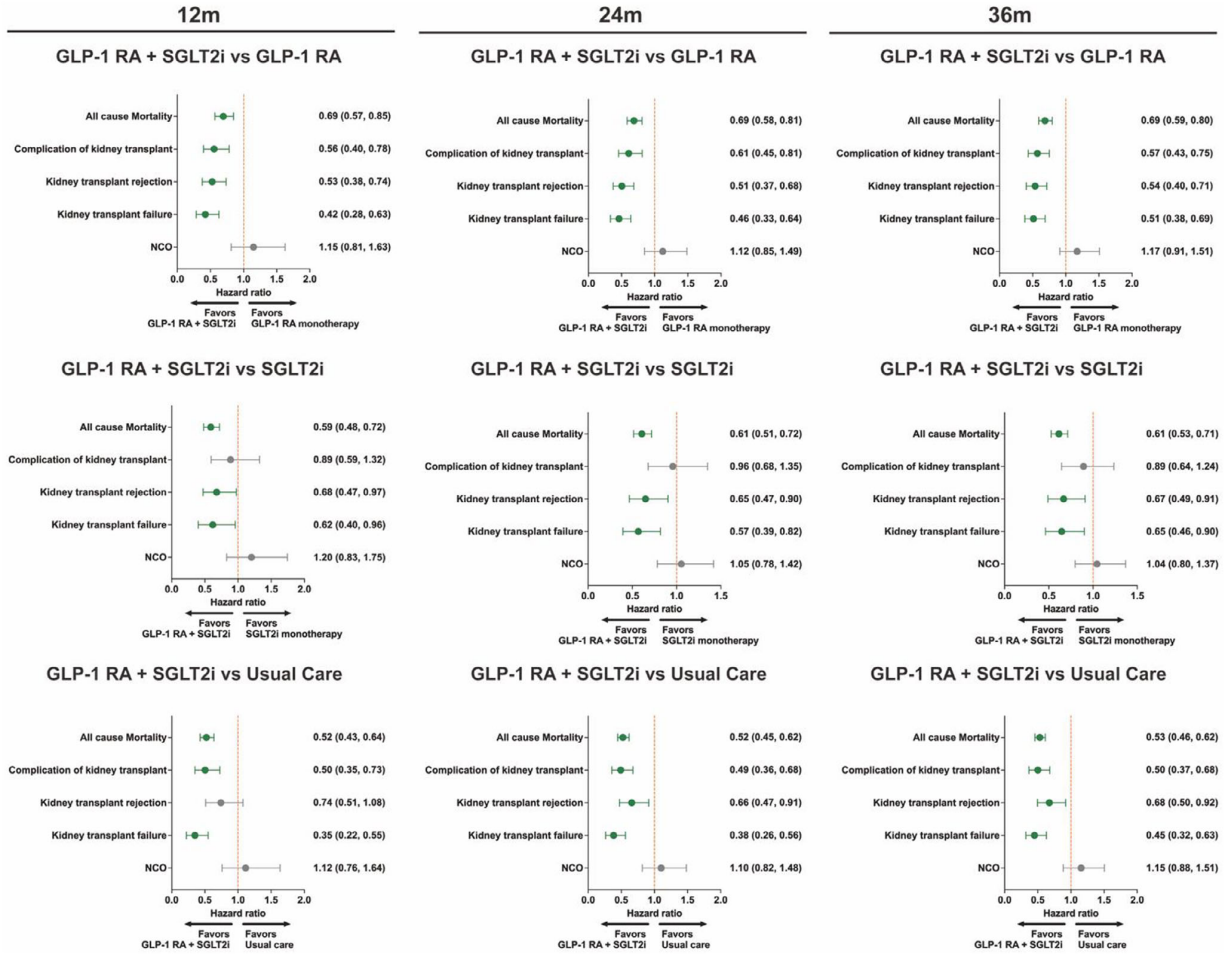
Forest plot analysis of transplant outcomes at 12‐, 24‐, and 36‐month. Forest plots displaying hazard ratios and 95% confidence intervals for transplant‐related outcomes across three time points: 12 months, 24 months, and 36 months. Each column represents a different comparison: dual GLP‐1 RA plus SGLT2i therapy versus GLP‐1 RA monotherapy, versus SGLT2i monotherapy, and versus usual care. Primary outcomes: all‐cause mortality, secondly outcomes including complications of kidney transplant, kidney transplant rejection, and kidney transplant failure. NCO represents negative control outcomes. Hazard ratios less than 1.0 favor dual therapy. All analyses were adjusted for baseline covariates using propensity score matching. GLP‐1 RA, glucagon‐like peptide‐1 receptor agonist; NCO, negative control outcome; SGLT2i, sodium‐glucose cotransporter‐2 inhibitor.

To assess durability beyond the first year, we examined using an intention‐to‐treat framework, the association of pre‐transplant dual therapy with post‐transplant mortality and kidney‐transplant endpoints (graft failure, rejection, and transplant complications) at 1, 2, and 3 years compared with GLP‐1 RA alone, SGLT2i alone, and usual care (**Tables** 2, 3, 4 and **Figure** **2**). Compared with GLP‐1 RA alone, dual‐therapy exposure was associated with lower all‐cause mortality (HR 0.69, 95% CI 0.57–0.85; *p* < 0.001), kidney‐transplant failure (0.42, 0.28–0.63; *p* < 0.001), rejection (0.53, 0.38–0.74; *p* < 0.001), and transplant complications (0.56, 0.40–0.78; *p* < 0.001). Versus SGLT2i alone, estimates were 0.59 (0.48–0.72; *p* < 0.001) for mortality, 0.62 (0.40–0.96; *p* = 0.046) for kidney‐transplant failure, 0.68 (0.47–0.97; *p* = 0.046) for rejection, and 0.89 (0.59–1.32; *p* = 0.558) for complications. Versus usual care, estimates were 0.52 (0.43–0.64; *p* < 0.001) for mortality, 0.35 (0.22–0.55; *p* < 0.001) for kidney‐transplant failure, 0.50 (0.35–0.73; *p* < 0.001) for complications, and 0.74 (0.51–1.08; *p* = 0.113) for rejection.

**Table 2:** Clinical outcomes, risk differences, and effect estimates for dual GLP‐1 RA plus SGLT2i therapy versus monotherapy and usual care at 12 months post‐transplantation.

	Exposure Cohort	Comparator Cohort	Risk Difference [95% CI]	ARD [%]	NNT	HR‐Based RRR [%]	HR [95% CI]	*P* value	Adjusted *p* value [BH Method]	E‐value for HR	E‐value [CI limit closest to null]
GLP‐1 RA + SGLT2i vs GLP‐1 RA monotherapy											
All‐cause mortality	160/ 4583 (0.035)	237/ 4557 (0.052)	−0.017 (‐0.025, ‐0.009)	−1.7	59	+28.81	0.69 (0.57, 0.85)	<0.001	<0.001	2.26	1.63
Complication of kidney transplant	51/ 3948 (0.013)	94/ 3976 (0.024)	−0.011 (‐0.017, ‐0.005)	−1.1	86	+44.44	0.56 (0.40, 0.78)	<0.001	<0.001	2.97	1.88
Kidney transplant rejection	52/ 4078 (0.013)	100/ 4071 (0.025)	−0.012 (‐0.018, ‐0.006)	−1.2	78	+46.43	0.53 (0.38, 0.74)	<0.001	<0.001	3.18	2.04
Kidney transplant failure	35/ 4114 (0.009)	84/ 4087 (0.021)	−0.012 (‐0.017, ‐0.007)	−1.2	75	+58.33	0.42 (0.28, 0.63)	<0.001	<0.001	4.19	2.55
NCO	67/ 4347 (0.015)	61/ 4360 (0.014)	0.001 (‐0.004, 0.006)	+0.1	N/A	N/A	1.15 (0.81, 1.63)	0.432	N/A	N/A	N/A
GLP‐1 RA + SGLT2i vs SGLT2i monotherapy											
All‐cause mortality	153/ 4160 (0.037)	253/ 4203 (0.060)	−0.023 (‐0.033, ‐0.014)	−2.3	36	+39.73	0.59 (0.48, 0.72)	<0.001	<0.001	2.78	2.12
Complication of kidney transplant	46/ 3618 (0.013)	50/ 3624 (0.014)	−0.001 (‐0.006, 0.004)	−0.1	N/A	N/A	0.89 (0.59, 1.32)	0.558	0.558	N/A	N/A
Kidney transplant rejection	50/ 3727 (0.013)	71/ 3733 (0.019)	−0.006 (‐0.011, 0.000)	−0.6	132	+30.43	0.68 (0.47, 0.97)	0.058	0.046	2.30	1.21
Kidney transplant failure	33/ 3742 (0.009)	52/ 3791 (0.014)	−0.005 (‐0.010, 0.000)	−0.5	150	+41.18	0.62 (0.40, 0.96)	0.058	0.046	2.61	1.25
NCO	62/ 3925 (0.016)	50/ 3922 (0.013)	0.003 (‐0.002, 0.008)	+0.3	N/A	N/A	1.20 (0.83, 1.75)	0.414	N/A	N/A	N/A
GLP‐1 RA + SGLT2i vs Usual Care											
All‐cause mortality	147/ 3681 (0.040)	296/ 3666 (0.081)	−0.041 (‐0.052, ‐0.030)	−4.1	26	+45.45	0.52 (0.43, 0.64)	<0.001	<0.001	3.26	2.50
Complication of kidney transplant	43/ 3278 (0.013)	88/ 3266 (0.027)	−0.014 (‐0.021, ‐0.007)	−1.4	68	+50.00	0.50 (0.35, 0.73)	<0.001	<0.001	3.41	2.08
Kidney transplant rejection	48/ 3347 (0.014)	67/ 3328 (0.020)	−0.006 (‐0.012, 0.000)	−0.6	N/A	N/A	0.74 (0.51, 1.08)	0.142	0.113	N/A	N/A
Kidney transplant failure	25/ 3371 (0.007)	74/ 3357 (0.022)	−0.015 (‐0.020, ‐0.009)	−1.5	63	+64.00	0.35 (0.22, 0.55)	<0.001	<0.001	5.16	3.04
NCO	54/ 3486 (0.015)	51/ 3464 (0.015)	0.001 (‐0.005, 0.007)	+0.1	N/A	N/A	1.12 (0.76, 1.64)	0.576	N/A	N/A	N/A

To complement relative hazards, we summarized clinical impact using 12‐month absolute risk differences derived from end‐point survival on the Kaplan–Meier scale, computing event risk as 1−S_KM (12 months) and reported numbers needed to treat are computed as NNT(t) = 1/|F _treat_(t)−F_control_(t)∣, where F_group_(t) is the cumulative incidence by time for that group, and are rounded upward to whole numbers; negative ARD indicates NNT for benefit, positive ARD indicates NNT for harm. Data presented as events/total patients (proportion). Risk differences calculated as dual therapy minus comparator group. HR‐based relative risk reduction (RRR) represents percentage change from baseline risk. Hazard ratios adjusted for baseline demographics, comorbidities, medications, and transplant characteristics using propensity score matching. *P* values adjusted using Benjamini‐Hochberg method to control false discovery rate across multiple comparisons. E‐values quantify robustness to unmeasured confounding; higher values indicate greater resistance to bias. NCO, negative control outcome. ARD, absolute risk difference; NNT, number needed to treat; BH, Benjamini‐Hochberg; CI, confidence interval; HR, hazard ratio; N/A, not applicable for non‐significant results.

**Table 3:** Clinical outcomes, risk differences, and effect estimates for dual GLP‐1 RA plus SGLT2i therapy versus monotherapy and usual care at 24 months post‐transplantation.

	Exposure cohort	Comparator cohort	Risk difference [95% CI]	ARD [%]	NNT	HR‐Based RRR [%]	HR [95% CI)	*p* value	Adjusted *p* value [BH Method]	E‐value for HR	E‐value [CI limit closest to null]
GLP‐1 RA + SGLT2i vs GLP‐1 RA monotherapy											
All‐cause mortality	238/ 4583 (0.052)	371/ 4557 (0.081)	−0.029 (‐0.040, ‐0.019)	−2.9	33	+28.85	0.69 (0.58, 0.81)	<0.001	<0.001	2.26	1.77
Complication of kidney transplant	72/ 3948 (0.018)	125/ 3976 (0.031)	−0.013 (‐0.020, ‐0.006)	−1.3	66	+37.50	0.61 (0.45, 0.81)	<0.001	<0.001	2.26	1.77
Kidney transplant rejection	62/ 4078 (0.015)	127/ 4071 (0.031)	−0.016 (‐0.023, ‐0.009)	−1.6	52	+51.28	0.51 (0.37, 0.68)	<0.001	<0.001	3.33	2.30
Kidney transplant failure	52/ 4114 (0.013)	118/ 4087 (0.029)	−0.016 (‐0.022, ‐0.010)	−1.6	51	+51.35	0.46 (0.33, 0.64)	<0.001	<0.001	3.77	2.50
NCO	99/ 4347 (0.023)	96/ 4360 (0.022)	0.001 (‐0.005, 0.007)	+0.1	N/A	N/A	1.12 (0.85, 1.49)	0.421	N/A	N/A	N/A
GLP‐1 RA + SGLT2i vs SGLT2i monotherapy											
All‐cause mortality	230/ 4160 (0.055)	366/ 4203 (0.087)	−0.032 (‐0.043, ‐0.021)	−3.2	23	+35.77	0.61 (0.51, 0.72)	<0.001	<0.001	2.78	2.12
Complication of kidney transplant	65/ 3618 (0.018)	65/ 3624 (0.018)	0.000 (‐0.006, 0.006)	0	N/A	N/A	0.96 (0.68, 1.35)	0.803	0.803	N/A	N/A
Kidney transplant rejection	59/ 3727 (0.016)	87/ 3733 (0.023)	−0.007 (‐0.014, ‐0.001)	−0.7	84	+37.50	0.65 (0.47, 0.90)	0.010	0.013	2.30	1.46
Kidney transplant failure	46/ 3742 (0.012)	78/ 3791 (0.021)	−0.008 (‐0.014, ‐0.003)	−0.8	70	+45.16	0.57 (0.39, 0.82)	<0.001	0.004	2.61	1.74
NCO	91/ 3925 (0.0123)	83/ 3922 (0.021)	0.002 (‐0.004, 0.009)	+0.2	N/A	N/A	1.05 (0.78, 1.42)	0.734	N/A	N/A	N/A
GLP‐1 RA + SGLT2i vs Usual Care											
All‐cause mortality	214/ 3681 (0.058)	463/ 3666 (0.126)	−0.068 (‐0.081, ‐0.055)	−6.8	16	+44.59	0.52 (0.45, 0.62)	<0.001	<0.001	2.26	1.63
Complication of kidney transplant	55/ 3278 (0.017)	121/ 3266 (0.037)	−0.020 (‐0.028, ‐0.012)	−2.0	44	+51.11	0.49 (0.36, 0.68)	<0.001	<0.001	2.97	1.88
Kidney transplant rejection	57/ 3347 (0.017)	94/ 3328 (0.028)	−0.011 (‐0.018, ‐0.004)	−1.1	78	+37.14	0.66 (0.47, 0.91)	0.012	0.012	3.18	2.04
Kidney transplant failure	36/ 3371 (0.011)	102/ 3357 (0.030)	−0.020 (‐0.026, ‐0.013)	−2.0	45	+59.46	0.38 (0.26, 0.56)	<0.001	<0.001	4.19	2.55
NCO	85/ 3486 (0.024)	89/ 3464 (0.026)	−0.001 (‐0.009, 0.006)	−0.1	N/A	N/A	1.10 (0.82, 1.48)	0.529	N/A	N/A	N/A

To complement relative hazards, we summarized clinical impact using 24‐month absolute risk differences derived from end‐point survival on the Kaplan–Meier scale, computing event risk as 1−S_KM (24 months) and reported numbers needed to treat are computed as NNT(t) = 1/|F _treat_(t)−F_control_(t)∣, where F_group_(t) is the cumulative incidence by time for that group, and are rounded upward to whole numbers; negative ARD indicates NNT for benefit, positive ARD indicates NNT for harm. Data presented as events/total patients (proportion). Risk differences calculated as dual therapy minus comparator group. HR‐based relative risk reduction (RRR) represents percentage change from baseline risk. Hazard ratios adjusted for baseline demographics, comorbidities, medications, and transplant characteristics using propensity score matching. *P* values adjusted using Benjamini‐Hochberg method to control false discovery rate across multiple comparisons. E‐values quantify robustness to unmeasured confounding; higher values indicate greater resistance to bias. NCO, negative control outcome. ARD, absolute risk difference; NNT, number needed to treat; BH, Benjamini‐Hochberg; CI, confidence interval; HR, hazard ratio; N/A, not applicable for non‐significant results.

**Table 4:** Clinical outcomes, risk differences, and effect estimates for dual GLP‐1 RA plus SGLT2i therapy versus monotherapy and usual care at 36 months post‐transplantation.

	Exposure cohort	Comparator cohort	Risk Difference [95% CI]	ARD [%]	NNT	HR‐Based RRR [%]	HR [95% CI]	*p* value	Adjusted *p* value [BH Method]	E‐value for HR	E‐value [CI limit closest to null]
GLP‐1 RA + SGLT2i vs GLP‐1 RA monotherapy											
All‐cause mortality	283/ 4583 (0.062)	462/ 4557 (0.101)	−0.040 (‐0.051, ‐0.028)	−4.0	23	+29.93	0.69 (0.59, 0.80)	<0.001	<0.001	2.26	1.81
Complication of kidney transplant	78/ 3948 (0.020)	148/ 3976 (0.037)	−0.017 (‐0.025, ‐0.010)	−1.7	42	+44.44	0.57 (0.43, 0.75)	<0.001	<0.001	2.90	2.00
Kidney transplant rejection	72/ 4078 (0.018)	142/ 4071 (0.035)	−0.017 (‐0.024, ‐0.010)	−1.7	49	+42.55	0.54 (0.40, 0.71)	<0.001	<0.001	3.11	2.17
Kidney transplant failure	65/ 4114 (0.016)	137/ 4087 (0.034)	−0.018 (‐0.024, ‐0.011)	−1.8	52	+39.58	0.51 (0.38, 0.69)	<0.001	<0.001	3.33	2.26
NCO	123/ 4347 (0.028)	120/ 4360 (0.028)	0.001 (‐0.006, 0.008)	+0.1	N/A	N/A	1.17 (0.91, 1.51)	0.218	N/A	N/A	N/A
GLP‐1 RA + SGLT2i vs SGLT2i monotherapy											
All‐cause mortality	273/ 4160 (0.066)	430/ 4203 (0.102)	−0.037 (‐0.049, ‐0.025)	−3.7	17	+34.30	0.61 (0.53, 0.71)	<0.001	<0.001	2.66	2.17
Complication of kidney transplant	70/ 3618 (0.019)	75/ 3624 (0.021)	−0.001 (‐0.008, 0.005)	−0.1	N/A	N/A	0.89 (0.64, 1.24)	0.491	0.491	N/A	N/A
Kidney transplant rejection	68/ 3727 (0.018)	97/ 3733 (0.026)	−0.008 (‐0.014, ‐0.001)	−0.8	71	+33.33	0.67 (0.49, 0.91)	0.010	0.013	2.35	1.43
Kidney transplant failure	58/ 3742 (0.015)	86/ 3791 (0.023)	−0.007 (‐0.013, ‐0.001)	−0.7	109	+23.68	0.65 (0.46, 0.90)	<0.001	0.013	2.45	1.46
NCO	110/ 3925 (0.028)	101/ 3922 (0.026)	0.002 (‐0.005, 0.009)	+0.2	N/A	N/A	1.04 (0.80, 1.37)	0.754	N/A	N/A	N/A
GLP‐1 RA + SGLT2i vs Usual Care											
All‐cause mortality	251/ 3681 (0.068)	577/ 3666 (0.157)	−0.089 (‐0.104, ‐0.075)	−8.9	12	+43.15	0.53 (0.46, 0.62)	<0.001	<0.001	3.18	2.61
Complication of kidney transplant	59/ 3278 (0.018)	131/ 3266 (0.040)	−0.022 (‐0.030, ‐0.014)	−2.2	41	+49.02	0.50 (0.37, 0.68)	<0.001	<0.001	3.41	2.30
Kidney transplant rejection	64/ 3347 (0.019)	108/ 3328 (0.032)	−0.013 (‐0.021, ‐0.006)	−1.3	69	+34.88	0.68 (0.50, 0.92)	0.012	0.012	2.30	1.39
Kidney transplant failure	47/ 3371 (0.014)	122/ 3357 (0.036)	−0.022 (‐0.030, ‐0.015)	−2.2	46	+45.83	0.45 (0.32, 0.63)	<0.001	<0.001	3.87	2.55
NCO	105/ 3486 (0.030)	115/ 3464 (0.033)	−0.003 (‐0.011, 0.005)	+0.3	N/A	N/A	1.15 (0.88, 1.51)	0.293	N/A	N/A	N/A

To complement relative hazards, we summarized clinical impact using 36‐month absolute risk differences derived from end‐point survival on the Kaplan–Meier scale, computing event risk as 1−S_KM (36 months) and reported numbers needed to treat are computed as NNT(t) = 1/|F _treat_(t)−F_control_(t)∣, where F_group_(t) is the cumulative incidence by time for that group, and are rounded upward to whole numbers; negative ARD indicates NNT for benefit, positive ARD indicates NNT for harm. Data presented as events/total patients (proportion). Risk differences calculated as dual therapy minus comparator group. HR‐based relative risk reduction (RRR) represents percentage change from baseline risk. Hazard ratios adjusted for baseline demographics, comorbidities, medications, and transplant characteristics using propensity score matching. *P* values adjusted using Benjamini‐Hochberg method to control false discovery rate across multiple comparisons. E‐values quantify robustness to unmeasured confounding; higher values indicate greater resistance to bias. NCO, negative control outcome. ARD, absolute risk difference; NNT, number needed to treat; BH, Benjamini‐Hochberg; CI, confidence interval; HR, hazard ratio; N/A, not applicable for non‐significant results

Relative to GLP‐1 RA alone, associations observed at 12 months persisted at two years: all‐cause mortality HR 0.69 (0.58–0.81; *p* < 0.001), kidney‐transplant failure 0.46 (0.33–0.64; *p* < 0.001), rejection 0.51 (0.37–0.68; *p* < 0.001), and transplant complications 0.61 (0.45–0.81; *p* < 0.001). At three years versus GLP‐1 RA alone, estimates remained directionally consistent: all‐cause mortality HR 0.69 (0.59–0.80; *p* < 0.001), kidney‐transplant failure 0.51 (0.38–0.69; *p* < 0.001), rejection 0.54 (0.40–0.71; *p* < 0.001), and transplant complications 0.57 (0.43–0.75; *p* < 0.001). Across the ITT horizons, the 12‐month associations were preserved through 24 and 36 months, indicating that the first‐year findings are consistent over longer follow‐up.

### 2.3 As‐Treated Early Post‐Transplant Dual Therapy Linked to Lower 1‐Year Mortality and Graft Events

We evaluated post‐transplant exposure using an as‐treated framework with three strata: no further therapy after the index transplant (isolating pre‐transplant optimization), continuation for 0–3 months, and continuation for 0–6 months (Figure S2, Supporting Information).

Across strata, dual therapy was associated with lower 1‐year all‐cause mortality versus each comparator (e.g., no post‐index versus GLP‐1 RA HR 0.58, 95% CI 0.42–0.79; *p* < 0.001; continuation 0–3 months versus GLP‐1 RA 0.50, 0.36–0.70; *p* < 0.001; continuation 0–6 months versus usual care 0.44, 0.33–0.58; *p* < 0.001). For kidney‐transplant failure, estimates favored dual therapy versus GLP‐1 RA across strata (no post‐index 0.32, 0.14–0.75; *p*  = 0.006; continuation 0–3 months 0.31, 0.12–0.77; *p*  = 0.008; continuation 0–6 months 0.36, 0.16–0.80; *p*  = 0.009) and versus usual care in the 0–6‐month stratum (0.39, 0.18–0.86; *p*  = 0.015), while contrasts versus SGLT2i were not statistically different. Composite rejection/failure similarly favored dual therapy versus GLP‐1 RA (no post‐index 0.43, 0.22–0.82; *p* = 0.008; continuation 0–3 months 0.46, 0.24–0.87; *p*  = 0.014; continuation 0–6 months 0.46, 0.25–0.85; *p*  = 0.010). The mortality association persisted at two and three years across strata and comparators. Patterns for kidney‐transplant endpoints at extended horizons were similar to the 12‐month results.

### 2.4 Infection‐Related Outcomes at 3, 6, and 12 Months Follow‐up

Secondary analyses evaluated infection‐related safety outcomes across the same time points and treatment comparisons in this immunocompromised population (Figure S3, Supporting Information).

At 3 months, dual therapy was associated with lower hazards of sepsis versus GLP‐1 RA (HR 0.70, 95% CI 0.51–0.95; *p* = 0.022) and versus usual care (0.58, 0.41–0.81; *p* = 0.001). Respiratory failure was lower versus GLP‐1 RA (0.62, 0.43–0.88; *p* = 0.007), SGLT2i (0.59, 0.41–0.84; *p* = 0.003), and usual care (0.52, 0.36–0.75; *p* < 0.001). Pneumonia was reduced versus usual care (0.60, 0.42–0.84; *p* = 0.003). At 6 months, reductions persisted for CMV disease versus GLP‐1 RA (0.69, 0.53–0.89; *p* = 0.004) and usual care (0.67, 0.51–0.88; *p* = 0.004). Sepsis was lower versus GLP‐1 RA (0.68, 0.53–0.87; *p* = 0.002), SGLT2i (0.74, 0.58–0.95; *p* = 0.017), and usual care (0.54, 0.41–0.70; *p* < 0.001). Respiratory failure decreased versus GLP‐1 RA (0.74, 0.56–0.97; *p* = 0.028), SGLT2i (0.67, 0.51–0.89; *p* = 0.006), and usual care (0.57, 0.43–0.76; *p* < 0.001). Pneumonia was lower versus usual care (0.57, 0.43–0.75; *p* < 0.001). At 12 months, CMV disease remained lower versus GLP‐1 RA (0.67, 0.54–0.84; *p* < 0.001) and usual care (0.68, 0.53–0.86; *p* < 0.001). Sepsis was reduced versus GLP‐1 RA (0.74, 0.61–0.90; *p* = 0.002), SGLT2i (0.82, 0.67–1.00; *p* = 0.049), and usual care (0.63, 0.51–0.77; *p* < 0.001). Respiratory failure was lower versus SGLT2i (0.80, 0.64–1.00; *p* = 0.049) and usual care (0.66, 0.53–0.83; *p* < 0.001), and pneumonia decreased versus usual care (0.63, 0.51–0.78; *p* < 0.001). Across time points, the directions of association were consistent with the main analysis.

### 2.5 Kaplan‐Meier Survival Analysis

**Figure 3:**
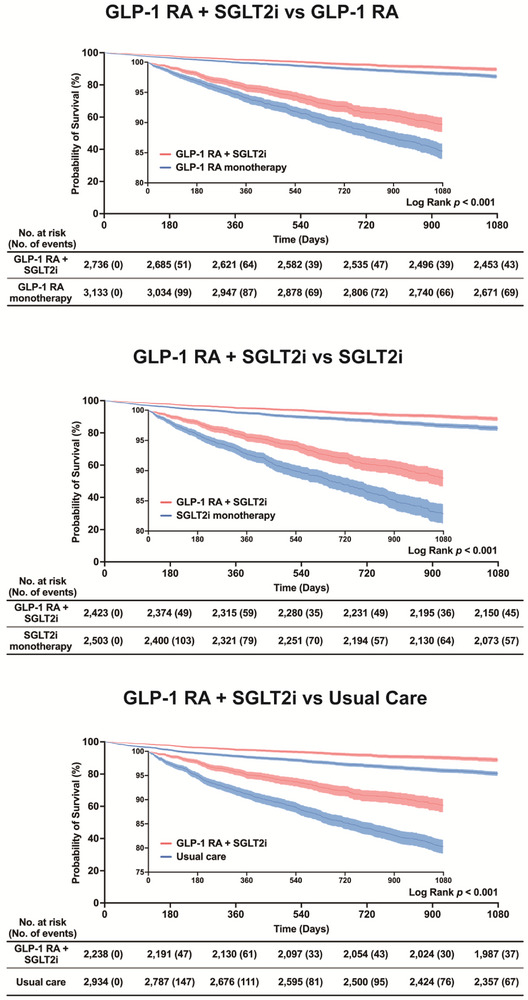
Kaplan‐Meier survival analysis of all‐cause mortality. Kaplan‐Meier survival curves demonstrating all‐cause mortality over 1080 days post‐transplantation across three treatment comparisons: (A) dual GLP‐1 RA plus SGLT2i therapy versus GLP‐1 RA monotherapy, (B) dual therapy versus SGLT2i monotherapy, and (C) dual therapy versus usual care. Numbers at risk and cumulative events are shown below each panel. All comparisons demonstrated statistically significant survival benefits favoring dual therapy (log‐rank *p* < 0.001). The inset panels show magnified views of the survival probability differences. GLP‐1 RA, glucagon‐like peptide‐1 receptor agonist; SGLT2i, sodium‐glucose cotransporter‐2 inhibitor.

Kaplan‐Meier curves indicated higher overall survival probabilities in the dual‐therapy cohort than in the GLP‐1 RA, SGLT2i, or usual‐care cohorts over the 1080‐day (3‐year) follow‐up period (**Figure** **3**). In each comparison, separation of the survival curves appeared within the first 6 months and was maintained through day 1080. Log‐rank tests with Benjamini–Hochberg correction yielded *p* < 0.001 for all three comparisons. The numbers at risk and cumulative event counts shown below each panel are consistent with fewer observed deaths in the dual‐therapy cohort and align with the hazard ratio estimates from the main analysis.

### 2.6 Organ‐Stratified Outcomes at 12, 24, and 36 Months

**Figure 4:**
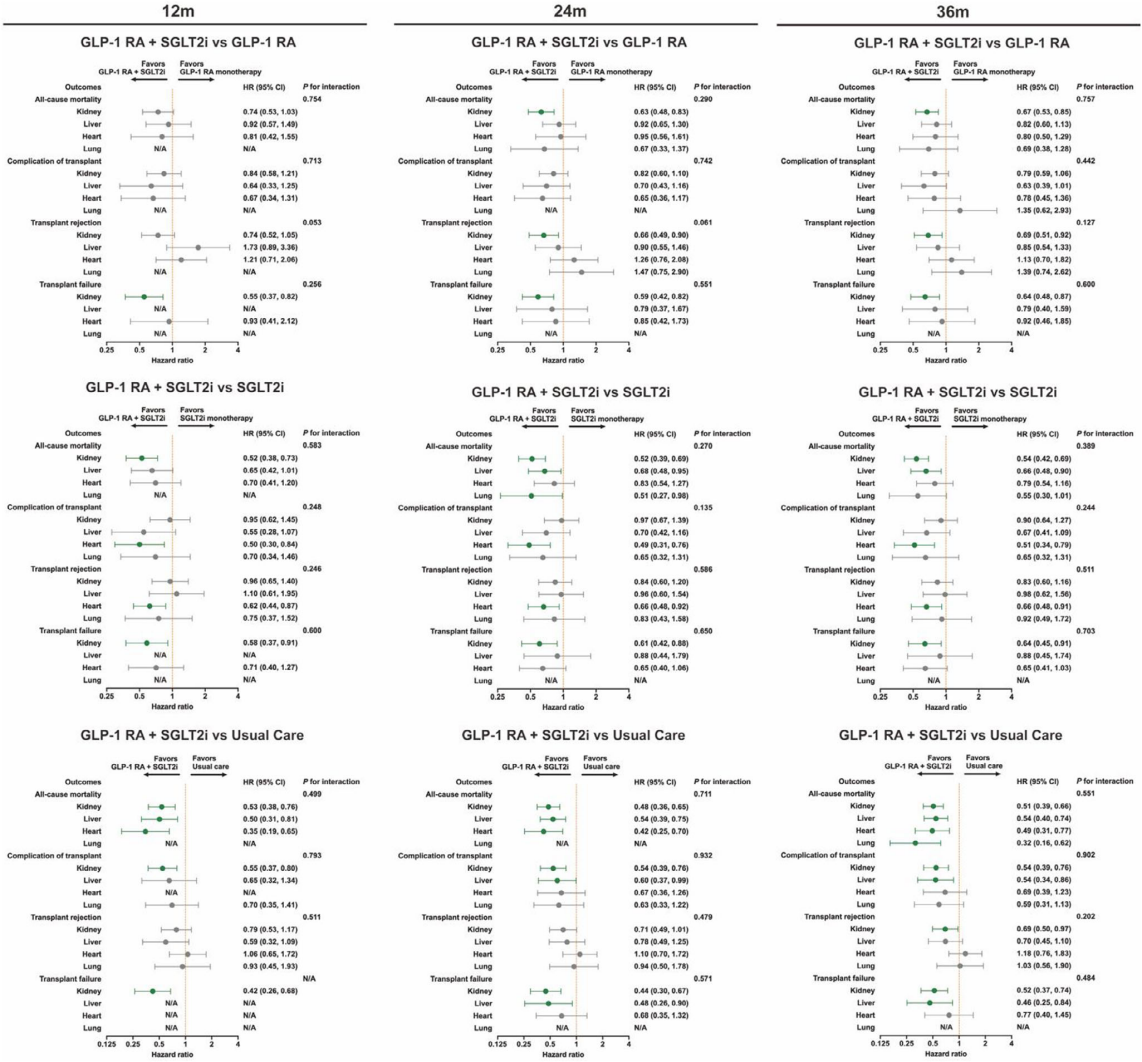
Organ‐stratified associations of pre‐transplant dual GLP‐1 receptor agonist plus SGLT2 inhibitor therapy with post‐transplant outcomes at 12, 24, and 36 months versus GLP‐1 RA monotherapy, SGLT2i monotherapy, and usual care. Forest plots display hazard ratios (points) with 95% confidence intervals (lines) from Cox models in 1:1 propensity score–matched cohorts, stratified by organ (kidney, liver, heart, lung) and by time horizon (12, 24, 36 months). Panels A–C compare dual therapy with GLP‐1 RA monotherapy, SGLT2i monotherapy, and usual care, respectively. The primary endpoint is all‐cause mortality in all organs. Values <1.0 favor dual therapy. *P* values for interaction are shown from treatment × organ terms at each horizon; non‐significant results indicate no evidence of effect modification by organ. “N/A” denotes non‐estimable cells due to sparse events. Models used prespecified demographics, comorbidities, medications, and laboratory covariates for matching. GLP‐1 RA, glucagon‐like peptide‐1 receptor agonist; SGLT2i, sodium–glucose cotransporter‐2 inhibitor; HR, hazard ratio; CI, confidence interval.

At 12 months, mortality associations favored dual therapy across organs, with no evidence of interaction (p for interaction: 0.754 vs GLP‐1 RA; 0.583 vs SGLT2i; 0.499 vs usual care; **Figure** **4**). At 24 months, interaction testing remained non‐significant (0.290; 0.270; 0.711), and associations persisted, notably in kidney (e.g., mortality 0.63 vs GLP‐1 RA; 0.52 vs SGLT2i; 0.48 vs usual care) and liver/heart/lung where estimable. At 36 months, interaction tests were again non‐significant (0.757; 0.389; 0.551), with sustained associations for kidney (mortality 0.67 vs GLP‐1 RA; 0.54 vs SGLT2i; 0.51 vs usual care; graft failure 0.64 vs GLP‐1 RA; 0.64 vs SGLT2i; 0.52 vs usual care) and favorable mortality estimates in liver (0.66 vs SGLT2i; 0.54 vs usual care), heart (0.49 vs usual care), and lung (0.32 vs usual care). For complications and rejection, interaction tests were non‐significant at all time points, with consistent signals in kidney (rejection vs GLP‐1 RA at 24–36 months) and heart (complications and rejection vs SGLT2i).

### 2.7 Safety Outcomes Analysis

**Figure 5:**
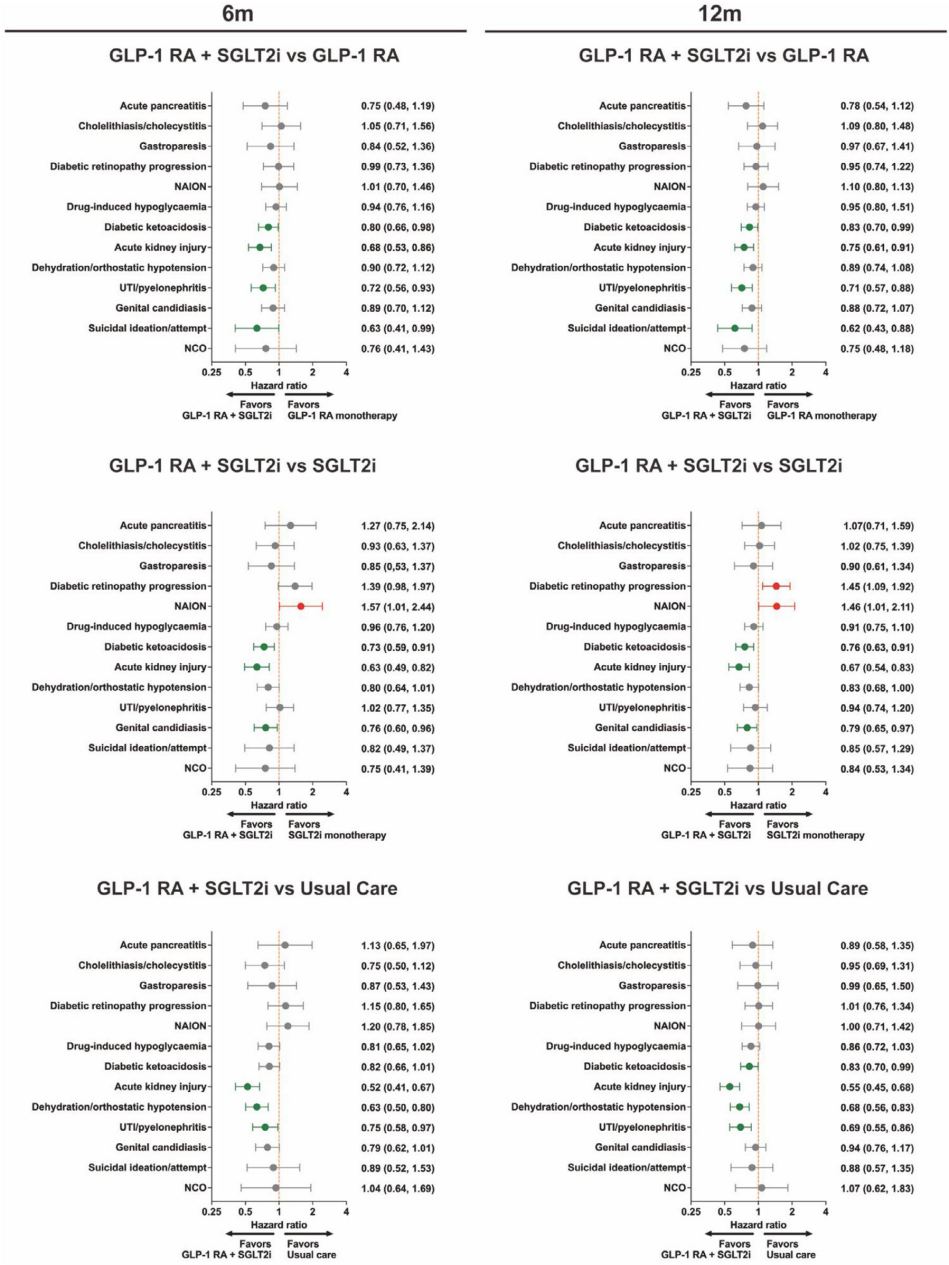
Safety outcomes at 6 and 12 months by treatment strategy. Forest plots of hazard ratios (95% CIs) for prespecified safety endpoints comparing pre‐transplant dual GLP‐1 RA plus SGLT2i with GLP‐1 RA monotherapy, SGLT2i monotherapy, and usual care at 6 months (left row) and 12 months (right row). Endpoints include acute pancreatitis; cholelithiasis/cholecystitis; gastroparesis; acute kidney injury (AKI); diabetic ketoacidosis (DKA); dehydration/orthostatic hypotension; urinary‐tract infection/pyelonephritis; genital candidiasis; hypoglycemia; diabetic retinopathy; nonarteritic anterior ischemic optic neuropathy (NAION); thyroid carcinoma; and suicidal ideation/attempt. Incident events were ascertained from ICD‐10‐CM codes; individuals with the diagnosis in baseline history were excluded from the respective analysis. Hazard ratios <1.0 favor dual therapy. GLP‐1 RA, glucagon‐like peptide‐1 receptor agonist; SGLT2i, sodium–glucose cotransporter‐2 inhibitor.

Safety profiles varied across treatment comparisons and follow‐up periods (**Figure** **5**). At 6 months, estimates for acute pancreatitis, cholelithiasis/cholecystitis, and gastroparesis were broadly null (e.g., pancreatitis vs GLP‐1 RA: HR 0.75, 95% CI 0.48–1.19; *p* = 0.221). Dual therapy showed lower associations with acute kidney injury versus GLP‐1 RA (0.68, 0.53–0.86; *p* = 0.001), SGLT2i (0.63, 0.49–0.82; *p* < 0.001), and usual care (0.52, 0.41–0.67; *p* < 0.001). Diabetic ketoacidosis also showed a lower association versus GLP‐1 RA (0.80, 0.66–0.98; *p* = 0.034) and SGLT2i (0.73, 0.59–0.91; *p* = 0.004), with a nonsignificant estimate versus usual care (0.82, 0.66–1.01; *p* = 0.067).

By 12 months, the pattern persisted. Pancreatitis remained null (e.g., vs GLP‐1 RA: 0.78, 0.54–1.12; *p* = 0.174). Dual therapy was associated with lower acute kidney injury versus GLP‐1 RA (0.75, 0.61–0.91; *p* = 0.003), SGLT2i (0.67, 0.54–0.83; *p* < 0.001), and usual care (0.55, 0.45–0.68; *p* < 0.001). Diabetic ketoacidosis showed lower associations versus GLP‐1 RA (0.83, 0.70–0.99; *p* = 0.037), SGLT2i (0.76, 0.63–0.91; *p* = 0.003), and usual care (0.83, 0.70–0.99; *p* = 0.042). Additional safety outcomes were favorable at 12 months, including dehydration/orthostatic hypotension versus usual care (0.68, 0.56–0.83; *p* < 0.001) and UTIs/pyelonephritis versus GLP‐1 RA (0.69, 0.55–0.86; *p* = 0.001).

### 2.8 Sensitivity Analysis

Geographic validation across North America, Europe, and Asia‐Pacific healthcare systems confirmed our primary findings (Figure S4, Supporting Information). In the pooled Global network, dual therapy was associated with lower all‐cause mortality versus GLP‐1 RA (HR 0.70, 95% CI 0.57–0.85; p < 0.001), SGLT2i (0.68, 0.56–0.83; p < 0.001), and usual care (0.55, 0.45–0.67; p < 0.001). Graft failure showed consistent reductions versus GLP‐1 RA (0.49, 0.33–0.71; p < 0.001) and usual care (0.29, 0.19–0.46; p < 0.001), with a borderline effect versus SGLT2i (0.65, 0.41–1.01; *p* = 0.053). Rejection decreased versus GLP‐1 RA and SGLT2i (both *p* ≤ 0.018), and overall complications were lower versus usual care (0.42, 0.29–0.60; p < 0.001). NCOs were null across contrasts. These results are consistent in direction with the main analysis.

Landmark analysis examined the 3–12 months period to assess sustained associations (Figure S5, Supporting Information). After applying a 3‐month landmark, dual therapy was associated with lower hazards versus GLP‐1 RA for all‐cause mortality (HR 0.76, 95% CI 0.60–0.96; *p* = 0.022), kidney‐transplant rejection (0.63, 0.41–0.97; *p* = 0.034), graft failure (0.43, 0.26–0.71; p < 0.001), and complications (0.55, 0.35–0.86; *p* = 0.009). Compared with SGLT2i, mortality (0.64, 0.51–0.82; p < 0.001) and graft failure (0.54, 0.31–0.94; *p* = 0.028) were lower, while rejection and complications were not different (both *p* > 0.23). Versus usual care, hazards were lower for mortality (0.60, 0.47–0.76; p < 0.001), complications (0.52, 0.30–0.82; *p* = 0.005), and graft failure (0.35, 0.19–0.65; p < 0.001), with no difference in rejection (*p* = 0.47); NCO remained null in 3‐group comparison. These findings are directionally consistent with the main analysis.

In early‐use sensitivity analyses limiting exposure assessment to the 12 or 6 months immediately preceding transplantation, estimated hazard ratios for all‐cause mortality and kidney‐graft outcomes were similar to the primary analysis (Figure S6, Supporting Information). Using a 12‐month window, the 1‐year HRs for all‐cause mortality with dual therapy versus GLP‐1 RA, SGLT2i, and usual care were 0.67 (95% CI 0.54–0.82; *p* < 0.001), 0.63 (0.52–0.77; *p* < 0.001), and 0.45 (0.36–0.57; *p* < 0.001), respectively; the corresponding HRs for kidney‐transplant failure were 0.45 (0.30–0.66; *p* < 0.001), 0.66 (0.42–1.03; *p* = 0.06), and 0.27 (0.16–0.44; *p* < 0.001). When the exposure window was restricted to 6 months, the 1‐year HRs for mortality were 0.70 (0.57–0.87; *p* = 0.001) versus GLP‐1 RA, 0.55 (0.45–0.68; *p* < 0.001) versus SGLT2i, and 0.43 (0.34–0.55; *p* < 0.001) versus usual care, and the HRs for kidney‐transplant failure were 0.41 (0.27–0.62; *p* < 0.001), 0.60 (0.38–0.95; *p* = 0.02), and 0.29 (0.17–0.48; *p* < 0.001), respectively (Figure S7, Supporting Information).

In the new‐user analysis with a 12‐month washout before treatment initiation, dual‐therapy new users were associated with lower hazards of mortality and kidney‐graft failure than SGLT2i new users and never users. The 1‐year mortality HRs were 0.64 (95% CI 0.52–0.79; *p* < 0.001) for dual therapy versus GLP‐1 RA, 0.56 (0.45–0.68; *p* < 0.001), versus SGLT2i and 0.41 (0.32–0.52; *p* < 0.001), versus usual care, and the 1‐year kidney‐transplant failure HRs were 0.46 (0.30–0.71; *p* < 0.001), 0.61 (0.39–0.94; *p* = 0.023) and 0.27 (0.16–0.46; *p* < 0.001), respectively (Figure S8, Supporting Information).

### 2.9 COVID‐Adjusted Analyses

After adding COVID‐19 covariates (Figure S9, Supporting Information), the 1‐year ITT estimates for dual therapy (GLP‐1 RA+SGLT2i) remained stable. Compared with GLP‐1 RA, adjusted hazard ratios (HRs) were 0.77 for all‐cause mortality (95% CI 0.63–0.94; *p* = 0.01), 0.53 for kidney‐transplant complications (0.38–0.75; *p* < 0.001), 0.56 for rejection (0.40–0.78; *p* < 0.001), and 0.45 for graft failure (0.31–0.66; *p* < 0.001). Versus SGLT2i, the HR for mortality was 0.61 (0.50–0.74; *p* < 0.001); kidney endpoints were directionally similar (complications 0.83 [0.54–1.25]; *p* = 0.365; rejection 0.73 [0.51–1.05]; *p* = 0.088; graft failure 0.67 [0.43–1.03]; *p* = 0.063). Versus usual care (metformin or sulfonylureas), HRs were 0.49 for mortality (0.40–0.59; *p* < 0.001), 0.41 for complications (0.28–0.62; *p* < 0.001), 0.55 for rejection (0.37–0.80; *p* = 0.002), and 0.32 for graft failure (0.20–0.50; *p* < 0.001). The NCO was null across comparisons (e.g., vs GLP‐1 RA: HR 1.10 [0.78–1.56]; *p* = 0.576). In calendar‐time sensitivity analyses restricted to 2016–2019, mortality estimates were comparable: HR 0.60 (0.49–0.74; *p* < 0.001) versus GLP‐1 RA, 0.68 (0.54–0.86; *p* = 0.002) versus SGLT2i, and 0.41 (0.32–0.52; *p* < 0.001) versus usual care.

### 2.10 Association of Post‐Transplant Dual Therapy with LDL‐C, CRP/NT‐proBNP, and HbA1c

We assessed post‐transplant biomarkers, HbA1c (%), BMI (kg m^−^^2^), weight (lb), LDL‐C (mg dL^−1^), CRP (mg L^−1^), and NT‐proBNP (pg mL^−1^)–‐at 0, 30, 60, 90, 180, and 360 days in the matched cohorts (Figure S10, Supporting Information).

At 360 days, compared with usual care, dual therapy showed lower CRP (38.82 vs 50.43 mg L^−1^; Δ −11.61; *p* = 0.01) and NT‐proBNP (2988.90 vs 4550.94 pg mL^−1^; Δ −1562.04; *p* = 0.04), while HbA1c (7.27 vs 7.03%; Δ +0.24; *p* < 0.001), weight (205.60 vs 202.77 lb; Δ +2.84; *p* = 0.021), and LDL‐C (82.88 vs 74.16 mg/dL; Δ +8.73; *p* < 0.001) were higher; the BMI difference was small (31.61 vs 31.40 kg m^−^^2^; Δ +0.21; *p* = 0.197). Significant differences across time included higher HbA1c at baseline and 60–360 days, higher LDL‐C at baseline and 90–360 days, higher weight at 30, 90, 180, and 360 days, higher BMI at 180 days, and lower CRP at 180 and 360 days; NT‐proBNP was lower at 360 days. Relative to SGLT2i monotherapy at 360 days, dual therapy had lower BMI (31.71 vs 32.15 kg m^−^^2^; Δ −0.45; *p* = 0.007) and LDL‐C (73.42 vs 77.72 mg dL^−1^; Δ −4.31; *p* < 0.001) with modestly higher HbA1c (7.30 vs 7.17%; Δ +0.13; *p* = 0.01). Over time, significant contrasts were higher HbA1c at 60–360 days, lower LDL‐C at 180 and 360 days, and lower BMI at 360 days.

Versus GLP‐1 RA monotherapy at 360 days, dual therapy showed lower LDL‐C (73.29 vs 79.85 mg dL^−1^; Δ −6.56; *p* < 0.001), alongside smaller reductions in BMI (31.22 vs 30.60 kg m^−^^2^; Δ +0.62; *p* < 0.001) and weight (204.04 vs 200.34 lb; Δ +3.71; *p* = 0.002), and slightly higher HbA1c (7.29 vs 7.12%; Δ +0.16; *p* = 0.001). Significant timepoint differences included lower LDL‐C at 30–360 days, higher HbA1c at 0, 60, 180, and 360 days, higher BMI at 0, 60, 90, 180, and 360 days, and higher weight at 0, 90, and 360 days.

## 3 Discussion

Our analysis of 15214 organ transplant candidates found that dual GLP‐1 RA and SGLT2i therapy before transplantation was associated with reduced mortality compared to monotherapy or usual care.

These results build on recent post‐transplant studies of individual agents. Orandi et al. reported 49% lower graft loss and 31% lower mortality with post‐transplant GLP‐1 RA initiation in 18016 kidney recipients.^[^ ^7^ ^]^ Our pre‐transplant dual approach showed larger effect sizes, which may reflect metabolic preparation before transplant‐related physiological stress. The safety profile aligns with broader evidence from high‐risk populations. Large‐scale trials have demonstrated cardiovascular protection with dual therapy in type 2 diabetes patients,^[^ ^11^ ^]^ while studies in obesity populations without diabetes showed cardiovascular benefits and acceptable safety profiles.^[^ ^17^, ^18^ ^]^ These findings in dialysis candidates and metabolically compromised patients provide relevant context for our transplant cohort.

Earlier small series in kidneytransplant recipients hinted that SGLT2 inhibition might increase genitourinary or systemic infections under chronic immunosuppression, prompting widespread caution in transplant practice.^[^ ^20^, ^21^, ^29^ ^]^ More recent multicenter and registry cohorts have not confirmed a clinically relevant excess risk,^[^ ^30^ ^]^ and an international electronichealthrecord analysis of more than 50 000 recipients showed infection rates that were neutral across all genitourinary and candidal endpoints.^[^ ^16^ ^]^ Consistent with these later observations, our pretransplant dualtherapy cohort demonstrated lower sepsis rates than each comparator group. Differences in exposure timing (preoperative metabolic optimization versus posttransplant rescue), baseline metabolic control, and the pleiotropic antiinflammatory effects attributed to both GLP‐1 receptor activation and SGLT2 blockade, ranging from endothelial nitricoxide enhancement to suppression of NF‐κB signaling, may underlie the divergent findings.^[^ ^31^, ^32^ ^]^

The magnitude of mortality reduction observed in our analysis exceeds the 12–15 % relative risk reductions reported in placebocontrolled cardiovascular outcome trials evaluating liraglutide, semaglutide, and dulaglutide in the broader population with type 2 diabetes.^[^ ^33^, ^34^ ^]^ Kidneytransplant candidates enter the perioperative period with a substantially higher burden of atherosclerotic disease and arrhythmic risk than participants enrolled in those trials.^[^ ^35^ ^]^ Observational registry data indicate that cardiovascular events account for up to one in three deaths on the waiting list, underscoring the larger scope for benefit that accompanies metabolic optimization before surgery.^[^ ^36^ ^]^

The target‐trial emulation framework strengthened internal validity by explicitly aligning eligibility criteria, exposure assignment, and follow‐up with those of a hypothetical randomized trial.^[^ ^24^ ^]^ Propensity‐score matching achieved excellent covariate balance, with all standardized mean differences below the widely accepted threshold of 0.10.^[^ ^37^ ^]^ Effect estimates remained directionally stable when we applied high‐dimensional propensity scores, and alternate caliper widths supporting the robustness of our findings.^[^ ^38^ ^]^ The multinational scope and large sample yielded power to detect clinically relevant differences that would escape smaller single‐center studies. Finally, by starting follow‐up 90 days after transplantation we used a landmark approach to minimizes immortal‐time bias.

The observed benefit likely reflects complementary mechanisms of GLP‐1 RAs and SGLT2i that converge on several pathways implicated in graft survival. GLP1 RAs potentiate glucosedependent insulin secretion and restrain inappropriate glucagon release, achieving durable glycemic control with minimal hypoglycemia.^[^ ^39^, ^40^ ^]^ Beyond metabolic effects, GLP1 receptor activation augments endothelial nitricoxide synthase activity, dampens systemic inflammation, and reprograms myocardial substrate use, changes associated with lower cardiovascular risk in outcome trials.^[^ ^41^ ^]^ SGLT2i provide distinct yet synergistic actions through glucosuriamediated calorie loss, natriuresisdriven volume contraction, and haematocrit elevation that improves oxygen delivery; preclinical work further shows attenuation of oxidative stress, suppression of NLRP3 inflammasome activation, and promotion of ketonebased energetics in cardiac and renal tissues.^[^ ^42^, ^43^ ^]^ Large randomized trials demonstrate that the resulting renoprotection is largely independent of glycaemic control, consistent with direct tubular effects and restoration of tubuloglomerular feedback.^[^ ^44^ ^]^

Metabolic preparation during kidney transplantation appears to blunt the peri‐operative catabolic surge, support tissue repair, and may permit lighter maintenance immunosuppression.^[^ ^45^, ^46^ ^]^ In our cohort, candidates who received a GLP‐1 RA together with a SGLT‐2i before surgery showed relative reduction in all‐cause mortality during the first post‐transplant year, accompanied by fewer septic events, findings consistent with an anti‐inflammatory effect. Existing guidelines still aim for an HbA1c below 7% but offer no direction on agent selection.^[^ ^47^ ^]^ The present results indicate that this dual‐agent strategy was associated with lower event rates than conventional regimens based on insulin, metformin, or sulfonylureas, and these associations became apparent early enough to be clinically meaningful even with a short pre‐transplant exposure.

Across three years of follow‐up, organ‐stratified analyses and interaction testing suggest broadly similar associations of pre‐transplant GLP‐1 RA+SGLT2i across transplant types, without statistical evidence of effect modification by organ. Estimates were most precise in kidney recipients, where kidney‐specific endpoints also favored dual therapy. Signals in liver, heart, and lung generally aligned with the overall pattern but were limited by smaller samples and sparse events in some organ–endpoint cells. Non‐estimable cells indicate insufficient events rather than opposing effects. These findings support the robustness of the main results and highlight the need for prospective confirmation, particularly in non‐kidney populations

Several inherent limitations constrain the interpretation of our findings. The observational design remains susceptible to unmeasured confounding despite sophisticated matching techniques. Provider selection biases may influence which patients receive dual therapy, potentially favoring those with better overall prognosis or more engaged healthcare behaviors. Medication adherence patterns, specific dosing protocols, and treatment duration were not uniformly captured across participating healthcare systems, limiting our ability to establish optimal therapeutic regimens or assess dose‐response relationships. We did not have serial laboratory data to describe kidney function trajectories (for example, estimated glomerular filtration rate or albuminuria), so kidney‐related findings in this study are restricted to clinical transplant events coded as graft failure, rejection, or complications. The electronic health record data source introduces potential coding inconsistencies and missing variables that could affect outcome ascertainment. Administrative coding may not fully capture the complexity of clinical decision‐making or patient‐specific factors that influence treatment selection.

Heterogeneity in transplant practices, immunosuppressive protocols, and patient care pathways across participating centers may limit generalizability to specific institutional settings. The study population derived from multinational healthcare networks may not reflect all transplant demographics, particularly underrepresented populations or healthcare systems with different resource availability. The specific agents used within each drug class, their relative market penetration, and prescribing preferences varied across regions and time periods, potentially influencing observed effects.

Long‐term follow‐up studies extending beyond one year are essential to determine whether early benefits translate into sustained graft survival improvements. The potential for delayed complications, such as diabetic retinopathy progression noted in some post‐transplant GLP‐1 RA studies, requires extended monitoring in pre‐transplant cohorts. Mechanistic studies examining biomarkers of inflammation, oxidative stress, and metabolic function could elucidate the pathways underlying observed benefits. Pharmacokinetic studies in transplant candidates would inform optimal dosing strategies, particularly given potential drug interactions with immunosuppressive agents.

The public health implications are substantial given the growing prevalence of diabetes and obesity among transplant candidates. Improved graft and patient survival could reduce healthcare costs, increase organ utilization efficiency, and enhance quality of life for thousands of patients annually. The potential to reduce infection rates in immunocompromised populations represents an additional public health benefit, particularly relevant in the era of antimicrobial resistance.

These findings also underscore the value of real‐world evidence generation using sophisticated analytical methods. Target‐trial emulation approaches may accelerate clinical knowledge development in areas where traditional randomized trials are challenging to conduct, providing timely evidence to guide clinical decision‐making while awaiting definitive trial results.

## 4 Experimental Section

### Ethics

This retrospective cohort investigation was approved by the Chung Shan Medical University Hospital Institutional Review Board (CS2‐24030). The investigation employed the TriNetX US Collaborative Network, a distributed research infrastructure that consolidates anonymized electronic medical records from more than 69 healthcare institutions throughout the United States using standardized data aggregation methods. TriNetX implements comprehensive anonymization protocols that surpass HIPAA Safe Harbor standards, incorporating sophisticated statistical techniques that automatically obscure small cohort sizes (1–10 subjects) to the nearest ten‐count increment, thereby preventing possible participant re‐identification. The platform completely prohibiting access to individual patient files or any personally identifying details. External privacy specialists have validated that these anonymization measures satisfy federal criteria for research exemption status. Due to the thorough anonymization of all data components and the inability to connect findings to specific individuals, this investigation met criteria for waiver of individual consent requirements according to 45 CFR 46.104(d)(4). The institutional review board determined that the study presented no greater than minimal risk to subjects and could not feasibly be executed using identifiable information. All investigational procedures followed ethical standards established in the Declaration of Helsinki concerning medical research with human participants.

### Target Trial Emulation and Design

Target trial emulation was adopted to address the limitations inherent in conventional observational analyses of pre‐transplant interventions.^[^ ^24^, ^48^ ^]^ Target trial emulation differs from standard retrospective cohort studies by first specifying the hypothetical randomized trial that would answer the research question, then mapping available observational data to approximate that trial's design.^[^ ^49^, ^50^ ^]^ This approach prevents common temporal biases, including immortal time bias and selection bias, which arise when eligibility determination and treatment assignment were not properly synchronized.^[^ ^51^ ^]^

Three factors motivated the choice of this methodology. Prospective randomized trials of pre‐transplant metabolic interventions face substantial ethical and practical constraints, particularly the narrow time window between listing and surgery.^[^ ^52^ ^]^ Standard retrospective analyses often suffer from temporal misalignment between exposure measurement and outcome assessment, creating artificial associations.^[^ ^16^ ^]^ The target trial framework forces explicit specification of design assumptions and temporal relationships, which proves essential when analyzing sustained treatment strategies.^[^ ^27^, ^53^ ^]^

Following established methodology, seven protocol components were specified: eligibility criteria, treatment strategies, assignment procedures, follow‐up period, outcomes, causal contrasts, and statistical analysis plan.^[^ ^27^ ^]^ Three separate pragmatic trials were emulated using 1:1 propensity score matching to compare pre‐transplant metabolic regimens (Table S1 and Figure S1, Supporting Information). Each comparison contrasted dual therapy (concurrent GLP‐1 RA and SGLT2i exposure within 24 months before surgery) against one alternative: GLP‐1 RA monotherapy, SGLT2i monotherapy, or usual care (absence of either study medication). Three analytical cohorts were constructed through 1:1 propensity score matching, comparing dual therapy against each alternative strategy. Usual care was operationalized as the absence of any GLP‐1 RA or SGLT2i prescriptions in the 24‐month pre‐index period, with metformin and/or sulfonylurea‐based management, and other non‐GLP‐1 RA/SGLT2i medications, per routine practice.

Treatment assignment reflected medication exposure patterns documented in electronic health records during the 24‐month pre‐transplant period. Propensity score matching created balanced treatment groups based on measured baseline characteristics, approximating the randomization process that would occur in an actual trial.^[^ ^26^, ^54^ ^]^ The transplantation date served as the index date, providing a fixed temporal reference for all analyses.^[^ ^55^ ^]^ This design explicitly defined eligibility criteria, treatment assignment periods, follow‐up protocols, and outcome ascertainment methods to minimize bias while approximating randomized trial conditions.^[^ ^56^ ^]^

### Data Source and Study Population

Analyses drew on the TriNetX US and Global Research Network, which compiles longitudinal, de‐identified electronic health records across North America, Europe, and Asia. Available data include demographics, diagnoses (ICD‐10‐CM), medications, procedures, and laboratory results dated January 1 2016 – August 31 2025. Individuals who underwent first solitary organ transplantation and had at least one year of continuous records before surgery were screened. Additional inclusion criteria were (i) type 2 diabetes documented by ICD‐10‐CM E11, with E66 or BMI ≥27 kg m^−2^ code during the 24 months before transplantation and (ii) no exposure to either study drug class during a 30‐day wash‐out. Among 608549 individuals with transplant status codes, kidney transplants represented the largest category (33.2%), followed by liver (14.7%) and other organ transplants (23.4% combined; Table S2, Supporting Information). After applying eligibility criteria for type 2 diabetes, obesity, and medication exposure, 15214 participants were included in the final analysis. Exclusion criteria comprised previous type 1 diabetes, HIV infection and prior bariatric surgery procedures (Table S3, Supporting Information). This study adhered to the STROBE (Strengthening the Reporting of Observational Studies in Epidemiology) reporting standards for observational research.

### Covariates

Baseline variables captured during the 12 months before transplantation included demographic characteristics (age, sex, race/ethnicity), anthropometric measures (body mass index), and comorbidity profiles encompassing chronic kidney disease, ischemic heart disease, atrial fibrillation and flutter, cerebral infarction, peripheral vascular diseases, chronic lower respiratory diseases, hyperlipidemia, hypertension, and mental and behavioral disorders due to psychoactive substance use. Healthcare utilization patterns were characterized by outpatient services and emergency department visits. Concomitant medications comprised antidiabetic agents (insulin, metformin, sulfonylureas, DPP‐4 inhibitors, thiazolidinediones), cardiovascular medications (HMG‐CoA reductase inhibitors, ACE inhibitors, angiotensin receptor blockers, beta‐blocking agents, antiarrhythmics, thiazides, aldosterone antagonists, aspirin), immunosuppressants, and antineoplastic agents. Laboratory parameters included creatinine levels, natriuretic peptide B, low‐density lipoprotein cholesterol, triglycerides, glycated hemoglobin A1c, alanine aminotransferase, and C‐reactive protein concentrations (Table S4, Supporting Information).

### Outcomes

Primary outcome was all‐cause mortality. Secondary outcomes comprised complication of kidney transplant, kidney transplant rejection, kidney transplant failure, and serious infection (cytomegaloviral, candidiasis, aspergillosis, mycoses, sepsis, pneumonia and respiratory failure). Safety surveillance focused on incident events: acute pancreatitis, cholelithiasis, gastroparesis, diabetic retinopathy, nonarteritic anterior ischemic optic neuropathy (NAION), thyroid carcinoma, hypoglycemia, diabetic ketoacidosis, dehydration, urinary‑tract infection (UTI), genital candidiasis and suicidal ideation/attempt. Outcomes were identified through ICD‐10‐CM code. Participants with any of these diagnoses during the history before cohort entry were excluded from the respective analysis to ensure incident‐event capture. Negative‐control outcomes (NCOs) were specified a priori to detect residual confounding, included other follicular disorders, radiculopathy of the lumbar region, conductive and sensorineural hearing loss, and skin cancer, selected a priori based on ref. [57] and their lack of a plausible causal relation with GLP‐1 RA or SGLT2i therapy (Table S5, Supporting Information). The primary analysis was performed at 12 months, with secondary extensions at 24 and 36 months. Each horizon was estimated separately using the same matched cohorts and censoring at the respective time point (Table S5, Supporting Information).

### Biomarkers

Post‐transplant biomarkers were evaluated in the same propensity score–matched cohorts and index date as the main analyses (index date = transplantation). Biomarkers were Hemoglobin A1c [HbA1c (%)], Body Mass Index [BMI, kg/m^−^^2^], weight (lb), LDL‐C (mg/dL^−1^), C‐reactive protein [CRP, mg/L^−1^], and N‐terminal pro‐B‐type natriuretic peptide [NT‐proBNP, pg/mL^−1^]. Measurements were summarized at nominal time points of 0, 30, 60, 90, 180, and 360 days after transplantation. Observed values were used without imputation. Analyses followed the intention‐to‐treat cohort assignment. For each contrast, we reported group means and mean differences (dual therapy–comparator) at each time point. Between‐group *p*‐values were derived from two‐sided independent‐samples t‐tests. Laboratory covariates with partial availability (such as BMI, HbA1c, and other prespecified markers) were analyzed as categorical variables with an added “Unavailable” level, and no imputation was applied. These *p*‐values are descriptive and were not adjusted for multiple comparisons (Table S5, Supporting Information).

### Statistical Analysis

The primary analytic horizon was 12 months after the index transplant. This interval captures the period of highest peri‐operative and immunologic risk and aligns with regulator‐facing benchmarks used for program performance reporting (e.g., first‐year graft and patient survival). To address concerns about durability beyond the first year, we pre‐specified two extended horizons at 24 and 36 months as secondary analyses.

In addition to the target‐trial emulation reported in the main analysis, we performed an intention to treat (ITT) re‐analysis. Exposure cohorts were assigned at the index date based on pre‐transplant treatment strategy (dual GLP‐1 RA+SGLT2i vs GLP‐1 RA alone, SGLT2i alone, or usual care). Under ITT, cohort assignment did not change with post‐index medication use. Follow‐up started on the transplant date and continued until the first of outcome occurrence, the end of the specified horizon (12, 24, or 36 months), end of capture, or loss to follow‐up. Propensity‐score matching procedures and model specifications were the same as in the primary analysis. We estimated hazard ratios (HRs) and 95% confidence intervals (CIs) using Cox models in the matched samples, with two‐sided α = 0.05. *p* values < 0.001 are reported as “<0.001.”

Baseline characteristics were presented as means with standard deviations for continuous data and frequencies with percentages for categorical variables. To minimize confounding, propensity score matching was implemented before conducting primary and sensitivity analyses. The TriNetX platform constructed a covariate matrix by extracting patient‐level variables from the year preceding the index date following cohort definition and outcome specification. Propensity scores representing the likelihood of assignment to each treatment group were generated through logistic regression modeling incorporating these baseline covariates. Patients underwent 1:1 matching using a greedy nearest‐neighbor algorithm with a caliper of 0.1 pooled standard deviations. Covariate balance was assessed using standardized mean differences (SMD), with values below 0.1 indicating satisfactory equilibrium between matched groups.^[^ ^58^ ^]^ Time‐to‐event analyses employed Cox proportional hazards regression to calculate hazard ratios and 95% confidence intervals. Survival distributions were compared using Kaplan‐Meier estimation with log‐rank testing. All statistical procedures were executed within the TriNetX analytical environment. To address multiple testing across principal comparisons, the false‐discovery rate was using the Benjamini–Hochberg procedure. E‐values quantified the influence of potential unmeasured confounders.^[^ ^59^ ^]^

For organ‐specific analyses and interaction testing, we conducted prespecified organ‐stratified analyses in kidney, liver, heart, and lung recipients using the same target‐trial emulation framework, covariates, and matching strategy as in the main analysis. Kidney‐specific endpoints (transplant complications, rejection, graft failure) were assessed only in kidney recipients. In the overall cohort, we fit Cox models including a treatment × organ interaction term to test for effect modification. Endpoints were summarized at 12, 24, and 36 months. We report stratum‐specific HRs with 95% confidence intervals and *p*‐values for interaction; “N/A” denotes event counts insufficient for reliable estimation (or suppressed per minimum cell‐size policy), and the corresponding comparisons were not modeled.

Several sensitivity analyses checks were undertaken to gauge the resilience of the principal findings. Landmark approach: to minimize immortal‐time and early postoperative bias, outcome surveillance began 90 days after transplantation; events recorded between T_0_ and day 90 were ignored, but follow‐up otherwise proceeded as in the primary analysis. Global collaborative network, which encompasses healthcare organizations from North America, Europe, and Asia‐Pacific. All scenarios employed Cox models parallel to the primary specification, and hazard ratios were contrasted with reference estimates to assess consistency.

In additional sensitivity analyses for immortal time, we progressively restricted the pre‐transplant exposure window and applied a new‐user design. First, treatment strategies were redefined using only medication exposure within the 12 months before transplant (−12 to −1 months) and, in a separate analysis, within the 6 months before transplant (−6 to −1 months), keeping the kidney‐transplant date as time zero. Second, a new‐user design is implemented with index at treatment, requiring at least 12 months of washout without GLP‐1 RA or SGLT2i before the first prescription of dual therapy, SGLT2i monotherapy, or usual care in the year before transplantation. For these new users, cohort membership was determined at first treatment initiation, but follow‐up for all outcomes remained anchored at the transplant date. All sensitivity analyses used the same covariates, propensity‐score matching, and outcome definitions as the primary ITT analysis.

Within the 24‐month pre‐transplant baseline window, we ascertained COVID‐19–related indicators and coded them as binary covariates: ICD‐10 U07.1 (COVID‐19 diagnosis), ICD‐10 U09.9 (post‐COVID condition), vaccination records (CVX 213), and SARS‐CoV‐2 testing codes (LOINC‐type 9088/9089 for RNA/antibody presence). These variables were added to the propensity‐score specification used for matching and retained in the outcome models under the intention‐to‐treat framework. As a calendar‐time sensitivity analysis, we repeated the main comparisons restricting the index period to 2016–2019 (pre‐pandemic).

Statistical analyses were conducted using the TriNetX platform, which integrates multiple computational environments. The analytical framework utilized Java 11.0.16 with Apache Commons Math 3.6.1 for core statistical operations, R 4.0.2 with Hmisc1‐1 and Survival 3.2‐3 packages for survival analysis, and Python 3.7 with lifelines, matplotlib, numpy, pandas, scipy, and statsmodels libraries for data manipulation and visualization.

## Conflict of Interest

The authors declare no conflict of interest.

## Author Contributions

Y.N.H. and T.H.T. conceived and designed the study; Y.N.H. conducted data analysis and wrote the first draft; P.H.L. and J.C.C. supervised all analyses and were responsible for data interpretation; M.Y.T., Y.L.L., and G.M.K. provided clinical expertise and critical revision. All authors participated in data acquisition and verification. P.H.L. and J.C.C. conducted external validation; Y.N.H. and M.Y.T. constructed the dataset using the TriNetX Global Research Network; Y.N.H., G.M.K., T.H.T., and P.H.S. reviewed manuscript drafts and provided substantive feedback. All authors had full access to the data, reviewed and approved the final manuscript, and take responsibility for the integrity of the work. T.H.T. and P.H.S. shared senior authorship (*Co‐corresponding authors).

## Supporting information



Supporting Information

## Data Availability

The data that support the findings of this study are available on request from the corresponding author. The data are not publicly available due to privacy or ethical restrictions.
