# Supplementary material for: Effectiveness of Pre‐Transplant Dual GLP‐1 Receptor Agonist and SGLT2 Inhibitor Therapy on All‐Cause Mortality in Organ Transplantation Candidates with Obesity and Type 2 Diabetes: a Target‐Trial Emulation
Source: Adv Sci (Weinh). 2025 Dec 12;13(11):e18813. doi: 10.1002/advs.202518813 (PMC12931237; doi:10.1002/advs.202518813)
Supplement: Supplementary file 1 — Supporting Information [file ADVS-13-e18813-s001.docx]

Supplementary material

Yu-Nan Huang^1,2^, Min-Yu Tsou^3^, Pin-Hung Li^1,2^, Jo-Ching Chen^1,2^, Yen-Liang Liu^3,4^, Gideon Meyerowitz-Katz^5^, Tsung-Hsun Tsai^6,^*, Pen-Hua Su^1,2,^*. **Effectiveness of Pre-transplant Dual GLP-1 Receptor Agonist and SGLT2 Inhibitor Therapy on All-Cause Mortality in Organ Transplantation Candidates with Obesity and Diabetes: A Target-Trial Emulation**

^1^Division of Genetics and Endocrinology, Department of Pediatrics, Chung Shan Medical University Hospital, Taichung, Taiwan; ^2^School of Medicine, Chung Shan Medical University; Taichung, Taiwan; ^3^Research Center for Cancer Biology, China Medical University, Taichung, Taiwan; ^4^Department of Medical Research, Chung Shan Medical University Hospital, Taichung, Taiwan; ^5^School of Health and Society, University of Wollongong, Wollongong, Australia; ^6^Division of Urology, Department of Surgery, Taichung Tzu Chi Hospital, Buddhist Tzu Chi Medical Foundation, Taichung, Taiwan. *Correspondence authors.

Correspondence to:

Tsung-Hsun Tsai, MD, PhD

Division of Urology, Department of Surgery, Taichung Tzu Chi Hospital, Buddhist Tzu Chi Medical Foundation, Taichung, Taiwan.

No. 88, Sec. 1, Fengxing Rd., Tanzi Dist., Taichung City 427, Taiwan (R.O.C.)

E-mail: [thh19760509@gmail.com](mailto:thh19760509@gmail.com)

Tel: +886 3606 0666 (Ext. 3456)

Pen-Hua Su, MD, PhD

Department Head and Professor

Division of Genetics and Metabolism, Department of Pediatrics, Chung Shan Medical University Hospital.

No. 110, Sec. 1, Jianguo N. Rd., South Dist., Taichung City 402306, Taiwan (R.O.C.)

E-mail: [ninaphsu@gmail.com](mailto:ninaphsu@gmail.com)

Tel: +886 4739595 (Ext. 21707)

Supplemental Online Content

Supplementary Table 1. Emulated target trial framework.

Supplementary Table 2. Organ Transplant Categories Among Study Participants.

Supplementary Table 3. Demographic, diagnostic, procedural, medication, visit, and laboratory codes used in the definition of the cohorts.

Supplementary Table 4. Demographic, diagnostic, laboratory, and medication codes used in the definition of covariates.

Supplementary Table 5. Diagnostic codes used in the definition of outcomes.

Supplementary Table 6. Baseline characteristics of organ transplant candidates with obesity and type 2 diabetes treated with dual GLP-1 RA plus SGLT2i versus SGLT2i monotherapy, before and after propensity score matching.

Supplementary Table 7. Baseline characteristics of organ transplant candidates with obesity and type 2 diabetes treated with dual GLP-1 RA plus SGLT2i versus usual care, before and after propensity score matching.

Supplementary **Figure 1. Target trial emulation design for pre-transplant dual metabolic therapy in organ transplant candidates**

Supplementary **Figure 2. As-treated post-transplant exposure strata (no post-index, 0–3 months, 0–6 months) and 12-month hazards for mortality and graft events comparing dual GLP-1 RA+SGLT2i with GLP-1 RA, SGLT2i, and usual care.**

**Supplementary Figure 3. Infection-related outcomes at 3, 6, and 12 months post-transplantation.**

**Supplementary Figure 4. Sensitivity analysis restricted to global collaborative network at 12 months.**

**Supplementary Figure 5. Landmark analysis of transplant outcomes.**

**Supplementary Figure 6. Early-use sensitivity analysis with a 12-month pre-transplant exposure window.**

**Supplementary Figure 7. Early-use sensitivity analysis with a 6-month pre-transplant exposure window.**

**Supplementary Figure 8. Index-at-treatment new-user analysis with a 12-month washout period.**

**Supplementary Figure 9. COVID-19–adjusted 12-month intention-to-treat estimates and pre-pandemic sensitivity, by comparator.**

**Supplementary Figure 10. Longitudinal post-transplant biomarkers through 360 days in matched cohorts (intention-to-treat).**

Supplementary Table 1. Emulated target trial framework

| Protocol component | Target trial | Emulated trial |
| --- | --- | --- |
| Aim | Compare risk of all-cause mortality among adults with type 2 diabetes listed for organ transplantation combined GLP‑1 RA + SGLT2i before surgery versus alternative metabolic strategies (GLP‑1 RA alone, SGLT2i alone, or usual care). | Same as the target trial. |
| Eligibility criteria | Adults aged ≥18 years with type 2 diabetes and obesity undergoing first organ transplantation; continuous records available for ≥24 months pre-transplant; 30-day washout period with no prior GLP-1 RA or SGLT2i exposure; exclusion of type 1 diabetes, HIV infection, multi-organ transplantation, and incomplete baseline data. | Same as the target trial – operationalized with TriNetX EHRs using ICD‑10‑CM codes, BMI ≥27 kg m² and medication dispense data. |
| Treatment strategies | Initiate dual GLP‑1 RA + SGLT2i therapy before transplant and continue through the peri‑operative period; comparators receive GLP‑1 RA alone, SGLT2i alone, or usual care. | Same as the target trial. |
| Treatment assignment | Random allocation with equal probability to each arm. | Individuals were non-randomly assigned to four treatment strategies based on real-world prescribing patterns during the pre-transplant period: dual GLP-1 RA plus SGLT2i therapy, GLP-1 RA monotherapy, SGLT2i monotherapy, or usual care without exposure to either study medication. Treatment assignment reflected clinical decision-making within the 24-month window before organ transplantation, with exposure defined as medication use during this period. Randomization was emulated through multinomial propensity score matching using a 1:1 nearest-neighbor algorithm across all four treatment arms. The matching incorporated baseline demographics, comorbidities, laboratory parameters, concomitant medications, and transplant-related factors measured during the year preceding surgery. The active comparator design with GLP-1 RA monotherapy or SGLT2i monotherapy. |
| Follow‑up | Starts on transplantation day (index) and ends on the occurrence of event, death, loss to follow‑up, or study end (12 months). | Start = transplant day; end = the occurrence of event, death, last record, or 31 Aug 2025. Landmark and Global region sensitivity analyses applied. |
| Outcomes | Primary: all‑cause mortality at 12 months. Secondary: acute kidney injury, major adverse cardiovascular events, serious infection and safety outcomes. | Same as the target trial. |
| Causal contrasts | Per‑protocol and intention‑to‑treat effects. | Primary analysis estimated intention‑to‑treat effect; sensitivity analyses estimated on‑treatment, Landmark and Global region effect. |
| Statistical analysis | Propensity‑score matching to create balanced cohorts; Kaplan–Meier estimation; Cox proportional‑hazards models to derive HRs; E‑values for unmeasured confounding; Benjamini–Hochberg control for multiple testing. | Same as the target trial. |

Supplementary Table 2. Organ Transplant Categories Among Study Participants

| Categories | Number | Percentage (%) |
| --- | --- | --- |
| Kidney transplant | 201,923 | 33.2 |
| Heart transplant | 33,579 | 5.5 |
| Lung transplant | 23,362 | 3.8 |
| Heart and lungs transplant | 1,427 | 0.2 |
| Liver transplant | 89,519 | 14.7 |
| Skin transplant | 53,772 | 8.8 |
| Bone transplant | 3,841 | 0.6 |
| Corneal transplant | 58,809 | 9.7 |
| Other transplanted organ and tissue | 116,006 | 19.1 |
| Transplanted organ and tissue, unspecified | 26,311 | 4.3 |

Distribution of organ transplant types among all participants identified with transplant status codes in the TriNetX database before applying study inclusion and exclusion criteria. Percentages represent the proportion of each transplant type within the total transplant population. Kidney transplants comprised the largest category (33.2%), followed by other/unspecified organ transplants (23.4% combined). The study population was subsequently filtered to include only participants meeting eligibility criteria for type 2 diabetes, obesity, and medication exposure requirements.

Supplementary Table 3. Demographic, diagnostic, procedural, medication, visit, and laboratory codes used in the definition of the cohorts.

| **Category** | **Code** | **Description** |
| --- | --- | --- |
| **SGLT2i + GLP-1 RA group** | | |
| **#1: At least 18 years old (must have the following).** | | |
| Demographics | Age | Age (at least 18 years) |
| **#1: Individuals with obesity and Type 2 diabetes treated with SGLT2i and GLP-1 RA (#1.3 must be fulfilled after #1.1 and #1.2). Date constraint: The terms in this group occurred on or before Aug 31, 2025.** | | |
| **#1.1: Individuals with obesity (must have any of the following).** | | |
| Diagnosis | UMLS:ICD10CM:E66 | Overweight and obesity |
| Laboratory | TNX:9083 | BMI (at least 27.00 kg/m2) |
| **#1.2: With type 2 diabetes (must have the following).** | | |
| Diagnosis | UMLS:ICD10CM:E11 | Type 2 diabetes mellitus |
| **#1.3: Receiving GLP-1 RA and SGLT2i (must have the following).** | | |
| Medication | NLM:ATC:A10BJ | Glucagon-like peptide-1 (GLP-1) analogues |
| Medication | NLM:ATC:A10BK | Sodium-glucose co-transporter 2 (SGLT2) inhibitors |
| **#2: With transplant surgery (must have the following). #2 must be fulfilled within 1 day after #1.3** | | |
| Diagnosis | UMLS:ICD10CM:Z94 | Transplanted organ and tissue status |
| Procedure | UMLS:SNOMED:77465005 | Transplantation |
| **#3: Without T1D, HIV, and bariatric surgery (cannot have any of the following). #3 must be fulfilled on or before #2** | | |
| Diagnosis | UMLS:ICD10CM:E10 | Type 1 diabetes mellitus |
| Diagnosis | UMLS:ICD10CM:B20 | Human immunodeficiency virus [HIV] disease |
| Procedure | UMLS:ICD10PCS:Z98.84 | Bariatric surgery status |
| Procedure | UMLS:CPT:1007385 | Bariatric surgery procedures |

| **GLP-1 RA group** | | |
| --- | --- | --- |
| **#1: At least 18 years old (must have the following).** | | |
| Demographics | Age | Age (at least 18 years) |
| **#1: Individuals with obesity and Type 2 diabetes treated with GLP-1 RA (#1.3 and #1.4 must be fulfilled after #1.1 and #1.2). Date constraint: The terms in this group occurred on or before Aug 31, 2025.** | | |
| **#1.1: Individuals with obesity (must have any of the following).** | | |
| Diagnosis | UMLS:ICD10CM:E66 | Overweight and obesity |
| Laboratory | TNX:9083 | BMI (at least 27.00 kg/m2) |
| **#1.2: With type 2 diabetes (must have the following).** | | |
| Diagnosis | UMLS:ICD10CM:E11 | Type 2 diabetes mellitus |
| **#1.3: Receiving GLP-1 RA (must have the following).** | | |
| Medication | NLM:ATC:A10BJ | Glucagon-like peptide-1 (GLP-1) analogues |
| **#1.4: Not receiving SGLT2i (cannot have the following)** | | |
| Medication | NLM:ATC:A10BK | Sodium-glucose co-transporter 2 (SGLT2) inhibitors |
| **#2: With transplant surgery (must have the following). #2 must be fulfilled within 1 day after #1.3** | | |
| Diagnosis | UMLS:ICD10CM:Z94 | Transplanted organ and tissue status |
| Procedure | UMLS:SNOMED:77465005 | Transplantation |
| **#3: Without T1D, HIV, and bariatric surgery (cannot have any of the following). #3 must be fulfilled on or before #2** | | |
| Diagnosis | UMLS:ICD10CM:E10 | Type 1 diabetes mellitus |
| Diagnosis | UMLS:ICD10CM:B20 | Human immunodeficiency virus [HIV] disease |
| Procedure | UMLS:ICD10PCS:Z98.84 | Bariatric surgery status |
| Procedure | UMLS:CPT:1007385 | Bariatric surgery procedures |

| **SGLT2i group** | | |
| --- | --- | --- |
| **#1: At least 18 years old (must have the following).** | | |
| Demographics | Age | Age (at least 18 years) |
| **#1: Individuals with obesity and Type 2 diabetes treated with SGLT2i (#1.3 and #1.4 must be fulfilled after #1.1 and #1.2). Date constraint: The terms in this group occurred on or before Aug 31, 2025.** | | |
| **#1.1: Individuals with obesity (must have any of the following).** | | |
| Diagnosis | UMLS:ICD10CM:E66 | Overweight and obesity |
| Laboratory | TNX:9083 | BMI (at least 27.00 kg/m2) |
| **#1.2: With type 2 diabetes (must have the following).** | | |
| Diagnosis | UMLS:ICD10CM:E11 | Type 2 diabetes mellitus |
| **#1.3: Receiving SGLT2i (must have the following).** | | |
| Medication | NLM:ATC:A10BK | Sodium-glucose co-transporter 2 (SGLT2) inhibitors |
| **#1.4: Not receiving GLP-1 RA (cannot have the following).** | | |
| Medication | NLM:ATC:A10BJ | Glucagon-like peptide-1 (GLP-1) analogues |
| **#2: With transplant surgery (must have the following). #2 must be fulfilled within 1 day after #1.3** | | |
| Diagnosis | UMLS:ICD10CM:Z94 | Transplanted organ and tissue status |
| Procedure | UMLS:SNOMED:77465005 | Transplantation |
| **#3: Without T1D, HIV, and bariatric surgery (cannot have any of the following). #3 must be fulfilled on or before #2** | | |
| Diagnosis | UMLS:ICD10CM:E10 | Type 1 diabetes mellitus |
| Diagnosis | UMLS:ICD10CM:B20 | Human immunodeficiency virus [HIV] disease |
| Procedure | UMLS:ICD10PCS:Z98.84 | Bariatric surgery status |
| Procedure | UMLS:CPT:1007385 | Bariatric surgery procedures |

| **Usual care group** | | |
| --- | --- | --- |
| **#1: At least 18 years old (must have the following).** | | |
| Demographics | Age | Age (at least 18 years) |
| **#1: Individuals with obesity and Type 2 diabetes treated with metformin or sulfonylurea (#1.3 and #1.4 must be fulfilled after #1.1 and #1.2). Date constraint: The terms in this group occurred on or before Aug 31, 2025.** | | |
| **#1.1: Individuals with obesity (must have any of the following).** | | |
| Diagnosis | UMLS:ICD10CM:E66 | Overweight and obesity |
| Laboratory | TNX:9083 | BMI (at least 27.00 kg/m2) |
| **#1.2: With type 2 diabetes (must have the following).** | | |
| Diagnosis | UMLS:ICD10CM:E11 | Type 2 diabetes mellitus |
| **#1.3: Receiving metformin or sulfonylurea (must have any of the following).** | | |
| Medication | NLM:RXNORM:6809 | metformin |
| Medication | NLM:ATC:A10BB | Sulfonylureas |
| **#1.4: Not receiving GLP-1 RA or SGLT2i (cannot have any of the following).** | | |
| Medication | NLM:ATC:A10BJ | Glucagon-like peptide-1 (GLP-1) analogues |
| Medication | NLM:ATC:A10BK | Sodium-glucose co-transporter 2 (SGLT2) inhibitors |
| **#2: With transplant surgery (must have the following). #2 must be fulfilled within 1 day after #1.3** | | |
| Diagnosis | UMLS:ICD10CM:Z94 | Transplanted organ and tissue status |
| Procedure | UMLS:SNOMED:77465005 | Transplantation |
| **#3: Without T1D, HIV, and bariatric surgery (cannot have any of the following). #3 must be fulfilled on or before #2** | | |
| Diagnosis | UMLS:ICD10CM:E10 | Type 1 diabetes mellitus |
| Diagnosis | UMLS:ICD10CM:B20 | Human immunodeficiency virus [HIV] disease |
| Procedure | UMLS:ICD10PCS:Z98.84 | Bariatric surgery status |
| Procedure | UMLS:CPT:1007385 | Bariatric surgery procedures |

Supplementary Table 4. Demographic, diagnostic, laboratory, and medication codes used in the definition of covariates.

| **Category** | **Code** | **Description** |
| --- | --- | --- |
| Demographics | AI | Age at index |
| Demographics | F | Female |
| Demographics | M | Male |
| Demographics | 2054-5 | Black or African American |
| Demographics | 2106-3 | White |
| Diagnosis | I10 | Essential (primary) hypertension |
| Diagnosis | E78.5 | Hyperlipidemia, unspecified |
| Diagnosis | I63 | Cerebral infarction |
| Diagnosis | I20-I25 | Ischemic heart diseases |
| Diagnosis | I48 | Atrial fibrillation and flutter |
| Diagnosis | N18 | Chronic kidney disease (CKD) |
| Diagnosis | I73 | Other peripheral vascular diseases |
| Diagnosis | J40-J4A | Chronic lower respiratory diseases |
| Diagnosis | F10-F19 | Mental and behavioral disorders due to psychoactive substance use |
| Diagnosis | Z55-Z65 | Persons with potential health hazards related to socioeconomic and psychosocial circumstances |
| Procedure | 1013626 | Office or Other Outpatient Services |
| Procedure | 1013711 | Emergency Department Services |
| Medication | C10AA | HMG CoA reductase inhibitors |
| Medication | C09AA | ACE inhibitors, plain |
| Medication | C09CA | Angiotensin II receptor blockers (ARBs), plain |
| Medication | C09DX | Angiotensin II receptor blockers (ARBs), other combinations |
| Medication | C07 | BETA BLOCKING AGENTS |
| Medication | C01B | ANTIARRHYTHMICS, CLASS I AND III |
| Medication | C03CA | Sulfonamides, plain |
| Medication | C03AA | Thiazides, plain |
| Medication | C03D | ALDOSTERONE ANTAGONISTS AND OTHER POTASSIUM-SPARING AGENTS |
| Medication | 1191 | aspirin |
| Medication | HS501 | INSULIN |
| Medication | 6809 | metformin |
| Medication | 4821 | glipizide |
| Medication | L01 | ANTINEOPLASTIC AGENTS |
| Medication | A10BA | Biguanides |
| Medication | A10BB | Sulfonylureas |
| Medication | A10BH | Dipeptidyl peptidase 4 (DPP-4) inhibitors |
| Medication | A10BX | Other blood glucose lowering drugs, excl. insulins |
| Medication | A10BG | Thiazolidinediones |
| Medication | L04 | IMMUNOSUPPRESSANTS |
| Medication | 213 | SARS-CoV-2 (COVID-19) Vaccine |
| Laboratory | 9083 | BMI |
| Diagnosis | 9024 | Creatinine [Mass/volume] in Serum, Plasma or Blood |
| Diagnosis | 9003 | Natriuretic peptide B [Mass/volume] in Serum, Plasma or Blood |
| Diagnosis | 9002 | Cholesterol in LDL [Mass/volume] in Serum or Plasma |
| Diagnosis | 9004 | Triglyceride [Mass/volume] in Serum, Plasma or Blood |
| Diagnosis | 9037 | Hemoglobin A1c/Hemoglobin.total in Blood |
| Diagnosis | 9044 | Alanine aminotransferase [Enzymatic activity/volume] in Serum, Plasma or Blood |
| Diagnosis | 9063 | C reactive protein [Mass/volume] in Serum, Plasma or Blood |
| Visit | EMER | Visit: Emergency |
| Visit | IMP | Visit: Inpatient Encounter |

Supplementary Table 5. Diagnostic codes used in the definition of outcomes.

| **Category** | **Code** | **Description** |
| --- | --- | --- |
| **#1**: **All-cause mortality** (have any of the following) | | |
| Demographics | Deceased | Deceased |
| Diagnosis | UMLS:ICD10CM:R99 | Ill-defined and unknown cause of mortality |
| **#2: Complication of kidney transplant** (have the following) | | |
| Diagnosis | UMLS:ICD10CM:T86.10 | Unspecified complication of kidney transplant |
| **#3: Kidney transplant rejection** (have the following) | | |
| Diagnosis | UMLS:ICD10CM:T86.11 | Kidney transplant rejection |
| **#4: Kidney transplant failure** (have the following) | | |
| Diagnosis | UMLS:ICD10CM:T86.12 | Kidney transplant failure |
| **#5: Complication of liver transplant** (have the following) | | |
| Diagnosis | UMLS:ICD10CM:T86.40 | Unspecified complication of liver transplant |
| **#6: Liver transplant rejection** (have the following) | | |
| Diagnosis | UMLS:ICD10CM:T86.41 | Liver transplant rejection |
| **#7: Liver transplant failure** (have the following) | | |
| Diagnosis | UMLS:ICD10CM:T86.42 | Liver transplant failure |
| **#8: Complication of heart transplant** (have the following) | | |
| Diagnosis | UMLS:ICD10CM:T86.20 | Unspecified complication of heart transplant |
| **#9: Heart transplant rejection** (have the following) | | |
| Diagnosis | UMLS:ICD10CM:T86.21 | Heart transplant rejection |
| **#10: Heart transplant failure** (have the following) | | |
| Diagnosis | UMLS:ICD10CM:T86.22 | Heart transplant failure |
| **#11: Complication of lung transplant** (have the following) | | |
| Diagnosis | UMLS:ICD10CM:T86.819 | Unspecified complication of lung transplant |
| **#12: Lung transplant rejection** (have the following) | | |
| Diagnosis | UMLS:ICD10CM:T86.810 | Lung transplant rejection |
| **#13: Lung transplant failure** (have the following) | | |
| Diagnosis | UMLS:ICD10CM:T86.811 | Lung transplant failure |
| **#14:** **Cytomegaloviral disease** (have the following) | | |
| Diagnosis | UMLS:ICD10CM:B25 | Cytomegaloviral disease |
| **#15: Candidiasis** (have the following) | | |
| Diagnosis | UMLS:ICD10CM:B37 | Candidiasis |
| **#16: Aspergillosis** (have the following) | | |
| Diagnosis | UMLS:ICD10CM:B44 | Aspergillosis |
| **#17: Mycoses** (have the following) | | |
| Diagnosis | UMLS:ICD10CM:B35-B49 | Mycoses |
| **#18: All-cause sepsis** (have any of the following) | | |
| Diagnosis | UMLS:ICD10CM:A40 | Streptococcal sepsis |
| Diagnosis | UMLS:ICD10CM:A41 | Other sepsis |
| **#19: All-cause pneumonia** (have any of the following) | | |
| Diagnosis | UMLS:ICD10CM:J12 | Viral pneumonia, not elsewhere classified |
| Diagnosis | UMLS:ICD10CM:J13 | Pneumonia due to Streptococcus pneumoniae |
| Diagnosis | UMLS:ICD10CM:J14 | Pneumonia due to Hemophilus influenzae |
| Diagnosis | UMLS:ICD10CM:J15 | Bacterial pneumonia, not elsewhere classified |
| Diagnosis | UMLS:ICD10CM:J16 | Pneumonia due to other infectious organisms, not elsewhere classified |
| Diagnosis | UMLS:ICD10CM:J17 | Pneumonia in diseases classified elsewhere |
| Diagnosis | UMLS:ICD10CM:J18 | Pneumonia, unspecified organism |
| **#20: Respiratory failure** (have the following) | | |
| Diagnosis | UMLS:ICD10CM:J96 | Respiratory failure, not elsewhere classified |
| **#21: Severe sepsis** (have the following) | | |
| Diagnosis | UMLS:ICD10CM:R65.2 | Severe sepsis |
| **#22: Acute pancreatitis** (have any of the following) | | |
| Diagnosis | UMLS:ICD10CM:K85 | Acute pancreatitis |
| Procedure | UMLS:CPT:74160 | Computed tomography, abdomen; with contrast material(s) |
| Procedure | UMLS:CPT:43264 | Endoscopic retrograde cholangiopancreatography (ERCP); with removal of calculi/debris from biliary/pancreatic duct(s) |
| **#23:** **Cholelithiasis/cholecystitis** (have any of the following) | | |
| Diagnosis | UMLS:ICD10CM:K80 | Cholelithiasis |
| Diagnosis | UMLS:ICD10CM:K81 | Cholecystitis |
| Procedure | UMLS:CPT:47562 | Laparoscopy, surgical; cholecystectomy |
| Procedure | UMLS:CPT:47563 | Laparoscopy, surgical; cholecystectomy with cholangiography |
| Procedure | UMLS:CPT:47564 | Laparoscopy, surgical; cholecystectomy with exploration of common duct |
| Procedure | UMLS:CPT:47480 | Cholecystotomy or cholecystostomy, open, with exploration, drainage, or removal of calculus (separate procedure) |
| **#24:** **Gastroparesis** (have any of the following) | | |
| Diagnosis | UMLS:ICD10CM:K31.84 | Gastroparesis |
| Procedure | UMLS:CPT:78264 | Gastric emptying imaging study (eg, solid, liquid, or both) |
| **#25:** **Diabetic retinopathy progression** (have any of the following) | | |
| Diagnosis | UMLS:ICD10CM:E11.3 | Type 2 diabetes mellitus with ophthalmic complications |
| Procedure | UMLS:CPT:67210 | Destruction of localized lesion of retina (eg, macular edema, tumors), 1 or more sessions; photocoagulation |
| Procedure | UMLS:CPT:67028 | Intravitreal injection of a pharmacologic agent (separate procedure) |
| **#26: NAION** (have any of the following) | | |
| Diagnosis | UMLS:ICD10CM:H47.01 | Ischemic optic neuropathy |
| Procedure | UMLS:CPT:92134 | Scanning computerized ophthalmic diagnostic imaging, posterior segment, with interpretation and report, unilateral or bilateral; retina |
| **#27: Drug‑induced hypoglycaemia** (have any of the following) | | |
| Diagnosis | UMLS:ICD10CM:E16.0 | Drug-induced hypoglycemia without coma |
| Diagnosis | UMLS:ICD10CM:E11.64 | Type 2 diabetes mellitus with hypoglycemia |
| Procedure | UMLS:CPT:96374 | Therapeutic, prophylactic, or diagnostic injection (specify substance or drug); intravenous push, single or initial substance/drug |
| Procedure | UMLS:CPT:96365 | Intravenous infusion, for therapy, prophylaxis, or diagnosis (specify substance or drug); initial, up to 1 hour |
| **#28:** **Diabetic ketoacidosis** (have any of the following) | | |
| Diagnosis | UMLS:ICD10CM:E11.1 | Type 2 diabetes mellitus with ketoacidosis |
| Diagnosis | UMLS:ICD10CM:E13.1 | Other specified diabetes mellitus with ketoacidosis |
| Diagnosis | UMLS:ICD10CM:E10.1 | Type 1 diabetes mellitus with ketoacidosis |
| Procedure | UMLS:CPT:99291 | Critical care, evaluation and management of the critically ill or critically injured patient; first 30-74 minutes |
| Procedure | UMLS:CPT:96365 | Intravenous infusion, for therapy, prophylaxis, or diagnosis (specify substance or drug); initial, up to 1 hour |
| **#29: Acute kidney injury** (have any of the following) | | |
| Diagnosis | UMLS:ICD10CM:N17 | Acute kidney failure |
| Procedure | UMLS:CPT:90935 | Hemodialysis procedure with single evaluation by a physician or other qualified health care professional |
| Procedure | UMLS:CPT:90945 | Dialysis procedure other than hemodialysis (eg, peritoneal dialysis, hemofiltration, or other continuous renal replacement therapies), with single evaluation by a physician or other qualified health care professional |
| **#30:** **Dehydration/orthostatic hypotension** (have any of the following) | | |
| Diagnosis | UMLS:ICD10CM:E86.0 | Dehydration |
| Diagnosis | UMLS:ICD10CM:I95.1 | Orthostatic hypotension |
| Procedure | UMLS:CPT:96360 | Intravenous infusion, hydration; initial, 31 minutes to 1 hour |
| **#31: UTI/pyelonephritis** (have any of the following) | | |
| Diagnosis | UMLS:ICD10CM:N39.0 | Urinary tract infection, site not specified |
| Diagnosis | UMLS:ICD10CM:N10 | Acute pyelonephritis |
| Procedure | UMLS:CPT:87086 | Culture, bacterial; quantitative colony count, urine |
| Procedure | UMLS:CPT:81001 | Urinalysis, by dip stick or tablet reagent for bilirubin, glucose, hemoglobin, ketones, leukocytes, nitrite, pH, protein, specific gravity, urobilinogen, any number of these constituents; automated, with microscopy |
| **#32:** **Genital candidiasis** (have any of the following) | | |
| Diagnosis | UMLS:ICD10CM:B37.49 | Other urogenital candidiasis |
| Diagnosis | UMLS:ICD10CM:B37.3 | Candidiasis of vulva and vagina |
| Procedure | UMLS:CPT:87205 | Smear, primary source with interpretation; Gram or Giemsa stain for bacteria, fungi, or cell types |
| **#33: Suicidal ideation / Attempt** (have any of the following) | | |
| Diagnosis | UMLS:ICD10CM:R45.851 | Suicidal ideations |
| Diagnosis | UMLS:ICD10CM:T14.91 | Suicide attempt |
| Procedure | UMLS:CPT:90791 | Psychiatric diagnostic evaluation |
| Procedure | UMLS:CPT:96127 | Brief emotional/behavioral assessment (eg, depression inventory, attention-deficit/hyperactivity disorder [ADHD] scale), with scoring and documentation, per standardized instrument |
| **#34: Negative control outcomes** | | |
| Diagnosis | UMLS:ICD10CM:L73 | Other follicular disorders |
| Diagnosis | UMLS:ICD10CM:M54.16 | Radiculopathy, lumbar region |
| Diagnosis | UMLS:ICD10CM:H90 | Conductive and sensorineural hearing loss |
| Diagnosis | UMLS:ICD10CM:C44 | Other and unspecified malignant neoplasm of skin |
| GlobalOncology | UMLS:ICDO3:C44 | Skin cancer |
| **#35: BMI** (have the following) | | |
| Laboratory | TNX:9083 | BMI |
| **#36: Weight** (have the following) | | |
| Laboratory | TNX:9081 | Body weight |
| **#37: HbA1c** (have the following) | | |
| Laboratory | TNX:9037 | Hemoglobin A1c/Hemoglobin.total in Blood (most recent occurrence) |
| **#38: LDL-C** (have the following) | | |
| Laboratory | TNX:9002 | Cholesterol in LDL [Mass/volume] in Serum or Plasma (most recent occurrence) |
| **#39: CRP** (have the following) | | |
| Laboratory | TNX:9063 | C reactive protein [Mass/volume] in Serum, Plasma or Blood ((most recent occurrence)) |
| **#40: NT-proBNP** (have the following) | | |
| Laboratory | TNX:9072 | Natriuretic peptide.B prohormone N-Terminal [Mass/volume] in Serum, Plasma or Blood ( (most recent occurrence)) |

Supplementary Table 6. Baseline characteristics of organ transplant candidates with obesity and type 2 diabetes treated with dual GLP-1 RA plus SGLT2i versus SGLT2i monotherapy, before and after propensity score matching

|  | Before Matching | | | After Matching | | |
| --- | --- | --- | --- | --- | --- | --- |
|  | GLP-1 RA +  SGLT2i | SGLT2i monotherapy | SMD | GLP-1 RA +  SGLT2i | SGLT2i monotherapy | SMD |
| Characteristic | (n = 5,230) | (n = 7,191) |  | (n = 4,282) | (n = 4,282) |  |
| **Age** |  |  |  |  |  |  |
| Mean ± SD | 59.9 ± 11.2 | 62.4 ± 11.4 |  | 60.6 ± 11.1 | 61.4 ± 11.5 |  |
| 0 - 20 years | 19 (0.4) | 21 (0.3) | 0.012 | 15 (0.3) | 12 (0.3) | 0.012 |
| 21 - 45 years | 533 (10.2) | 574 (8.0) | 0.077 | 388 (9.1) | 393 (9.2) | 0.004 |
| 46 - 65 years | 2,896 (55.4) | 3,482 (48.4) | 0.139 | 2,285 (53.4) | 2,260 (52.8) | 0.012 |
| ≥ 66 years | 1,782 (34.1) | 3,114 (43.3) | 0.190 | 1,594 (37.2) | 1,617 (37.8) | 0.011 |
| **Sex (%)** |  |  |  |  |  |  |
| Male | 3,269 (62.5) | 4,978 (69.2) | 0.142 | 2,790 (65.2) | 2,775 (64.8) | 0.007 |
| Female | 1,902 (36.4) | 2,098 (29.2) | 0.154 | 1,439 (33.6) | 1,456 (34.0) | 0.008 |
| **Race (%)** |  |  |  |  |  |  |
| White | 3,000 (57.4) | 4,034 (56.1) | 0.026 | 2,431 (56.8) | 2,441 (57.0) | 0.005 |
| Black or African American | 1,246 (23.8) | 1,666 (23.2) | 0.015 | 1,019 (23.8) | 1,012 (23.6) | 0.004 |
| **Socioeconomic determinants** |  |  |  |  |  |  |
| Persons with potential health hazards related to socioeconomic and psychosocial circumstances | 318 (6.1) | 487 (6.8) | 0.028 | 269 (6.3) | 269 (6.3) | <0.001 |
| **Measures of healthcare utilization** |  |  |  |  |  |  |
| Visit: inpatient encounter | 2,647 (50.6) | 4,005 (55.7) | 0.102 | 2,303 (53.8) | 2,322 (54.2) | 0.009 |
| Visit: emergency | 1,972 (37.7) | 2,613 (36.3) | 0.028 | 1,590 (37.1) | 1,608 (37.6) | 0.009 |
| Office or other outpatient services | 3,975 (76.0) | 5,478 (76.2) | 0.004 | 3,272 (76.4) | 3,228 (75.4) | 0.024 |
| Emergency department services | 2,106 (40.3) | 2,947 (41.0) | 0.015 | 1,758 (41.1) | 1,761 (41.1) | 0.001 |
| **Comorbidities** |  |  |  |  |  |  |
| Hypertension | 4,594 (87.8) | 5,930 (82.5) | 0.152 | 3,724 (87.0) | 3,708 (86.6) | 0.011 |
| Hyperlipidemia | 3,666 (70.1) | 4,707 (65.5) | 0.099 | 2,963 (69.2) | 2,945 (68.8) | 0.009 |
| CKD | 3,481 (66.6) | 4,658 (64.8) | 0.038 | 2,834 (66.2) | 2,821 (65.9) | 0.006 |
| IHD | 2,283 (43.7) | 3,455 (48.0) | 0.088 | 1,960 (45.8) | 1,960 (45.8) | <0.001 |
| Chronic lower respiratory diseases | 1,242 (23.7) | 1,671 (23.2) | 0.012 | 1,018 (23.8) | 1,042 (24.3) | 0.013 |
| Atrial fibrillation and flutter | 992 (19.0) | 1,876 (26.1) | 0.171 | 905 (21.1) | 885 (20.7) | 0.011 |
| Mental and behavioral disorders due to psychoactive substance use | 830 (15.9) | 1,211 (16.8) | 0.026 | 699 (16.3) | 700 (16.3) | <0.001 |
| Peripheral vascular diseases | 536 (10.2) | 812 (11.3) | 0.034 | 459 (10.7) | 467 (10.9) | 0.006 |
| Cerebral infarction | 291 (5.6) | 470 (6.5) | 0.041 | 260 (6.1) | 269 (6.3) | 0.009 |
| **Medications** |  |  |  |  |  |  |
| Beta-blocking agents | 3,580 (68.5) | 5,137 (71.4) | 0.065 | 2,996 (70.0) | 3,010 (70.3) | 0.007 |
| ACE inhibitors | 1,401 (26.8) | 1,713 (23.8) | 0.068 | 1,111 (25.9) | 1,116 (26.1) | 0.003 |
| ARBs | 2,013 (38.5) | 2,977 (41.4) | 0.059 | 1,658 (38.7) | 1,627 (38.0) | 0.015 |
| ARBs, other combinations | 341 (6.5) | 802 (11.2) | 0.164 | 319 (7.5) | 292 (6.8) | 0.024 |
| Thiazides, plain | 740 (14.1) | 981 (13.6) | 0.015 | 599 (14.0) | 594 (13.9) | 0.003 |
| Aldosterone antagonists and other potassium-sparing agents | 1,059 (20.2) | 1,877 (26.1) | 0.139 | 938 (21.9) | 943 (22.0) | 0.003 |
| Antiarrhythmics, class I and III | 3,449 (65.9) | 4,924 (68.5) | 0.054 | 2,893 (67.6) | 2,948 (68.8) | 0.028 |
| HMG-CoA reductase inhibitors | 4,077 (78.0) | 5,062 (70.4) | 0.173 | 3,262 (76.2) | 3,292 (76.9) | 0.017 |
| Biguanides | 2,069 (39.6) | 2,144 (29.8) | 0.206 | 1,555 (36.3) | 1,568 (36.6) | 0.006 |
| Sulfonylureas | 951 (18.2) | 1,013 (14.1) | 0.112 | 735 (17.2) | 725 (16.9) | 0.006 |
| DPP-4 inhibitors | 830 (15.9) | 1,009 (14.0) | 0.052 | 692 (16.2) | 676 (15.8) | 0.010 |
| Thiazolidinediones | 231 (4.4) | 151 (2.1) | 0.131 | 140 (3.3) | 129 (3.0) | 0.015 |
| Immunosuppressants | 3,479 (66.5) | 4,581 (63.7) | 0.059 | 2,837 (66.3) | 2,867 (67.0) | 0.015 |
| Sulfonamides | 2,675 (51.1) | 4,157 (57.8) | 0.134 | 2,317 (54.1) | 2,329 (54.4) | 0.006 |
| Antineoplastic agents | 1,057 (20.2) | 1,444 (20.1) | 0.003 | 871 (20.3) | 892 (20.8) | 0.012 |
| Insulin | 4,190 (80.1) | 4,694 (65.3) | 0.338 | 3,293 (76.9) | 3,310 (77.3) | 0.009 |
| Metformin | 2,069 (39.6) | 2,144 (29.8) | 0.206 | 1,555 (36.3) | 1,568 (36.6) | 0.006 |
| Glipizide | 631 (12.1) | 625 (8.7) | 0.111 | 470 (11.0) | 472 (11.0) | 0.001 |
| Aspirin | 2,516 (48.1) | 3,497 (48.6) | 0.010 | 2,109 (49.3) | 2,131 (49.8) | 0.010 |
| COVID-19 vaccine | 1,053 (20.1) | 1,207 (16.8) | 0.086 | 800 (18.7) | 779 (18.2) | 0.013 |
| **Laboratory** |  |  |  |  |  |  |
| **BMI** |  |  |  |  |  |  |
| Mean ± SD, kg/m^2^ | 32.4 ± 6.0 | 30.4 ± 5.6 |  | 32.1 ± 6.0 | 30.9 ± 5.6 |  |
| ≥ 30 kg/m^2^ | 3,689 (70.5) | 4,369 (60.8) | 0.207 | 2,900 (67.7) | 2,896 (67.6) | 0.002 |
| **ALT** |  |  |  |  |  |  |
| Mean ± SD, U/L | 29.4 ± 87.4 | 29.9 ± 101.0 |  | 30.0 ± 96.5 | 30.1 ± 94.8 |  |
| ≥ 30 U/L | 2,705 (51.7) | 3,675 (51.1) | 0.012 | 2,209 (51.6) | 2,224 (51.9) | 0.007 |
| **Creatinine** |  |  |  |  |  |  |
| Mean ± SD, mg/dL | 1.4 ± 1.6 | 1.5 ± 1.3 |  | 1.5 ± 1.8 | 1.5 ± 1.3 |  |
| ≥ 1.5 mg/dL | 2,586 (49.4) | 3,887 (54.1) | 0.092 | 2,209 (51.6) | 2,191 (51.2) | 0.008 |
| **HbA1C** |  |  |  |  |  |  |
| Mean ± SD, % | 7.6 ± 1.7 | 7.1 ± 1.6 |  | 7.5 ± 1.6 | 7.4 ± 1.6 |  |
| ≥ 7 % | 3,076 (58.8) | 3,048 (42.4) | 0.333 | 2,319 (54.2) | 2,322 (54.2) | 0.001 |
| **TG** |  |  |  |  |  |  |
| Mean ± SD, mg/dL | 181.8 ± 148.3 | 162.0 ± 122.9 |  | 174.1 ± 134.0 | 171.8 ± 121.5 |  |
| ≥ 200 u/L | 1,652 (31.6) | 1,683 (23.4) | 0.184 | 1,237 (28.9) | 1,224 (28.6) | 0.007 |
| **LDL-C** |  |  |  |  |  |  |
| Mean ± SD, mg/dL | 76.7 ± 33.9 | 79.2 ± 36.0 |  | 76.5 ± 33.7 | 79.2 ± 34.8 |  |
| ≥ 190 mg/dL | 76 (1.5) | 99 (1.4) | 0.006 | 62 (1.4) | 56 (1.3) | 0.012 |
| **CRP** |  |  |  |  |  |  |
| Mean ± SD, mg/L | 36.7 ± 61.9 | 36.0 ± 55.3 |  | 37.4 ± 62.6 | 34.7 ± 53.2 |  |
| ≥ 3 mg/L | 823 (15.7) | 1,302 (18.1) | 0.063 | 714 (16.7) | 739 (17.3) | 0.016 |
| **BNP** |  |  |  |  |  |  |
| Mean ± SD, pg/mL | 428.6 ± 1355.8 | 815.3 ± 2494.6 |  | 467.9 ± 1443.8 | 614.7 ± 1872.0 |  |
| ≥ 100 pg/mL | 649 (12.4) | 1,193 (16.6) | 0.119 | 590 (13.8) | 576 (13.5) | 0.010 |

GLP-1 RA, glucagon-like peptide-1 receptor agonist; SGLT2, sodium-glucose cotransporter 2; SMD, standardized mean difference; IHD, ischemia heart disease; CKD, chronic kidney disease; HMG-CoA, 3-hydroxy-3-methylglutaryl coenzyme A; ACE inhibitors, angiotensin-converting enzyme inhibitors; ARBs, angiotensin II receptor blockers; DPP-4 inhibitors, dipeptidyl peptidase-4 inhibitors; COVID-19, coronavirus disease 2019; BMI, body mass index; HbA1c, glycated hemoglobin A1c; LDL-C, low-density lipoprotein cholesterol; ALT, alanine aminotransferase; TG, triglycerides; CRP, C-reactive protein; BNP, B-type natriuretic peptide. Percentages are rounded to one decimal place. SMD is rounded to two decimal places.

Supplementary Table 7. Baseline characteristics of organ transplant candidates with obesity and type 2 diabetes treated with dual GLP-1 RA plus SGLT2i versus usual care, before and after propensity score matching

|  | Before Matching | | | After Matching | | |
| --- | --- | --- | --- | --- | --- | --- |
|  | GLP-1 RA +  SGLT2i | Usual care | SMD | GLP-1 RA +  SGLT2i | Usual care | SMD |
| Characteristic | (n = 5,230) | (n = 18,340) |  | (n = 3,787) | (n = 3,787) |  |
| **Age** |  |  |  |  |  |  |
| Mean ± SD | 59.9 ± 11.2 | 61.7 ± 12.4 |  | 60.2 ± 11.3 | 60.9 ± 12.0 |  |
| 0 - 20 years | 19 (0.4) | 118 (0.6) | 0.040 | 18 (0.5) | 19 (0.5) | 0.004 |
| 21 - 45 years | 533 (10.2) | 1,722 (9.4) | 0.027 | 368 (9.7) | 399 (10.5) | 0.027 |
| 46 - 65 years | 2,896 (55.4) | 9,016 (49.2) | 0.125 | 2,043 (53.9) | 2,027 (53.5) | 0.008 |
| ≥ 66 years | 1,782 (34.1) | 7,484 (40.8) | 0.139 | 1,358 (35.9) | 1,342 (35.4) | 0.009 |
| **Sex (%)** |  |  |  |  |  |  |
| Male | 3,269 (62.5) | 11,199 (61.1) | 0.030 | 2,341 (61.8) | 2,375 (62.7) | 0.019 |
| Female | 1,902 (36.4) | 6,832 (37.3) | 0.018 | 1,399 (36.9) | 1,367 (36.1) | 0.018 |
| **Race (%)** |  |  |  |  |  |  |
| White | 3,000 (57.4) | 11,321 (61.7) | 0.089 | 2,218 (58.6) | 2,237 (59.1) | 0.010 |
| Black or African American | 1,246 (23.8) | 3,511 (19.1) | 0.114 | 852 (22.5) | 844 (22.3) | 0.005 |
| **Socioeconomic determinants** |  |  |  |  |  |  |
| Persons with potential health hazards related to socioeconomic and psychosocial circumstances | 318 (6.1) | 669 (3.6) | 0.113 | 182 (4.8) | 185 (4.9) | 0.004 |
| **Measures of healthcare utilization** |  |  |  |  |  |  |
| Visit: inpatient encounter | 2,647 (50.6) | 10,503 (57.3) | 0.134 | 1,952 (51.5) | 1,937 (51.1) | 0.008 |
| Visit: emergency | 1,972 (37.7) | 5,735 (31.3) | 0.136 | 1,337 (35.3) | 1,354 (35.8) | 0.009 |
| Office or other outpatient services | 3,975 (76.0) | 12,486 (68.1) | 0.177 | 2,820 (74.5) | 2,811 (74.2) | 0.005 |
| Emergency department services | 2,106 (40.3) | 6,159 (33.6) | 0.139 | 1,437 (37.9) | 1,471 (38.8) | 0.018 |
| **Comorbidities** |  |  |  |  |  |  |
| Hypertension | 4,594 (87.8) | 14,302 (78.0) | 0.264 | 3,239 (85.5) | 3,213 (84.8) | 0.019 |
| Hyperlipidemia | 3,666 (70.1) | 9,559 (52.1) | 0.375 | 2,469 (65.2) | 2,494 (65.9) | 0.014 |
| CKD | 3,481 (66.6) | 8,445 (46.0) | 0.423 | 2,277 (60.1) | 2,276 (60.1) | <0.001 |
| IHD | 2,283 (43.7) | 5,860 (32.0) | 0.243 | 1,504 (39.7) | 1,472 (38.9) | 0.017 |
| Chronic lower respiratory diseases | 1,242 (23.7) | 3,836 (20.9) | 0.068 | 868 (22.9) | 869 (22.9) | <0.001 |
| Atrial fibrillation and flutter | 992 (19.0) | 2,696 (14.7) | 0.114 | 615 (16.2) | 643 (17.0) | 0.020 |
| Mental and behavioral disorders due to psychoactive substance use | 830 (15.9) | 3,076 (16.8) | 0.024 | 618 (16.3) | 581 (15.3) | 0.027 |
| Peripheral vascular diseases | 536 (10.2) | 1,399 (7.6) | 0.092 | 350 (9.2) | 361 (9.5) | 0.010 |
| Cerebral infarction | 291 (5.6) | 772 (4.2) | 0.063 | 186 (4.9) | 205 (5.4) | 0.023 |
| **Medications** |  |  |  |  |  |  |
| Beta-blocking agents | 3,580 (68.5) | 11,387 (62.1) | 0.134 | 2,492 (65.8) | 2,496 (65.9) | 0.002 |
| ACE inhibitors | 1,401 (26.8) | 5,412 (29.5) | 0.061 | 1,085 (28.7) | 1,088 (28.7) | 0.002 |
| ARBs | 2,013 (38.5) | 3,934 (21.4) | 0.379 | 1,201 (31.7) | 1,263 (33.4) | 0.035 |
| ARBs, other combinations | 341 (6.5) | 144 (0.8) | 0.309 | 107 (2.8) | 120 (3.2) | 0.020 |
| Thiazides, plain | 740 (14.1) | 2,837 (15.5) | 0.037 | 526 (13.9) | 563 (14.9) | 0.028 |
| Aldosterone antagonists and other potassium-sparing agents | 1,059 (20.2) | 2,427 (13.2) | 0.189 | 634 (16.7) | 673 (17.8) | 0.027 |
| Antiarrhythmics, class I and III | 3,449 (65.9) | 11,192 (61.0) | 0.102 | 2,423 (64.0) | 2,421 (63.9) | 0.001 |
| HMG-CoA reductase inhibitors | 4,077 (78.0) | 10,526 (57.4) | 0.451 | 2,784 (73.5) | 2,825 (74.6) | 0.025 |
| Biguanides | 2,069 (39.6) | 10,030 (54.7) | 0.307 | 1,809 (47.8) | 1,880 (49.6) | 0.038 |
| Sulfonylureas | 951 (18.2) | 7,268 (39.6) | 0.487 | 910 (24.0) | 975 (25.7) | 0.040 |
| DPP-4 inhibitors | 830 (15.9) | 2,095 (11.4) | 0.130 | 585 (15.4) | 612 (16.2) | 0.020 |
| Thiazolidinediones | 231 (4.4) | 631 (3.4) | 0.050 | 172 (4.5) | 188 (5.0) | 0.020 |
| Immunosuppressants | 3,479 (66.5) | 8,842 (48.2) | 0.377 | 2,315 (61.1) | 2,297 (60.7) | 0.010 |
| Sulfonamides | 2,675 (51.1) | 8,656 (47.2) | 0.079 | 1,865 (49.2) | 1,869 (49.4) | 0.002 |
| Antineoplastic agents | 1,057 (20.2) | 3,918 (21.4) | 0.028 | 762 (20.1) | 805 (21.3) | 0.028 |
| Insulin | 4,190 (80.1) | 11,226 (61.2) | 0.424 | 2,852 (75.3) | 2,865 (75.7) | 0.008 |
| Metformin | 2,069 (39.6) | 10,030 (54.7) | 0.307 | 1,809 (47.8) | 1,880 (49.6) | 0.038 |
| Glipizide | 631 (12.1) | 4,676 (25.5) | 0.349 | 598 (15.8) | 621 (16.4) | 0.017 |
| Aspirin | 2,516 (48.1) | 7,695 (42.0) | 0.124 | 1,750 (46.2) | 1,774 (46.8) | 0.013 |
| COVID-19 vaccine | 1,053 (20.1) | 1,194 (6.5) | 0.409 | 514 (13.6) | 547 (14.4) | 0.025 |
| **Laboratory** |  |  |  |  |  |  |
| **BMI** |  |  |  |  |  |  |
| Mean ± SD, kg/m^2^ | 32.4 ± 6.0 | 31.4 ± 6.3 |  | 32.3 ± 6.2 | 31.5 ± 6.2 |  |
| ≥ 30 kg/m^2^ | 3,689 (70.5) | 11,692 (63.8) | 0.145 | 2,573 (67.9) | 2,567 (67.8) | 0.003 |
| **ALT** |  |  |  |  |  |  |
| Mean ± SD, U/L | 29.4 ± 87.4 | 31.7 ± 58.4 |  | 30.4 ± 100.4 | 29.6 ± 45.7 |  |
| ≥ 30 U/L | 2,705 (51.7) | 8,756 (47.7) | 0.080 | 1,880 (49.6) | 1,905 (50.3) | 0.013 |
| **Creatinine** |  |  |  |  |  |  |
| Mean ± SD, mg/dL | 1.4 ± 1.6 | 1.7 ± 2.2 |  | 1.4 ± 1.9 | 1.8 ± 2.1 |  |
| ≥ 1.5 mg/dL | 2,586 (49.4) | 7,993 (43.6) | 0.118 | 1,769 (46.7) | 1,830 (48.3) | 0.032 |
| **HbA1C** |  |  |  |  |  |  |
| Mean ± SD, % | 7.6 ± 1.7 | 7.0 ± 1.7 |  | 7.5 ± 1.7 | 7.3 ± 1.7 |  |
| ≥ 7 % | 3,076 (58.8) | 6,833 (37.3) | 0.442 | 2,043 (53.9) | 2,082 (55.0) | 0.021 |
| **TG** |  |  |  |  |  |  |
| Mean ± SD, mg/dL | 181.8 ± 148.3 | 172.0 ± 140.6 |  | 178.8 ± 145.5 | 182.2 ± 159.8 |  |
| ≥ 200 u/L | 1,652 (31.6) | 3,724 (20.3) | 0.260 | 1,065 (28.1) | 1,104 (29.2) | 0.023 |
| **LDL-C** |  |  |  |  |  |  |
| Mean ± SD, mg/dL | 76.7 ± 33.9 | 84.6 ± 37.6 |  | 77.2 ± 34.3 | 82.6 ± 36.5 |  |
| ≥ 190 mg/dL | 76 (1.5) | 228 (1.2) | 0.018 | 54 (1.4) | 54 (1.4) | <0.001 |
| **CRP** |  |  |  |  |  |  |
| Mean ± SD, mg/L | 36.7 ± 61.9 | 41.9 ± 65.5 |  | 38.8 ± 66.7 | 40.4 ± 65.4 |  |
| ≥ 3 mg/L | 823 (15.7) | 3,135 (17.1) | 0.037 | 590 (15.6) | 598 (15.8) | 0.006 |
| **BNP** |  |  |  |  |  |  |
| Mean ± SD, pg/mL | 428.6 ± 1355.8 | 639.4 ± 2514.5 |  | 473.6 ± 1638.8 | 855.7 ± 3488.8 |  |
| ≥ 100 pg/mL | 649 (12.4) | 1,796 (9.8) | 0.083 | 392 (10.4) | 428 (11.3) | 0.031 |

GLP-1 RA, glucagon-like peptide-1 receptor agonist; SGLT2, sodium-glucose cotransporter 2; SMD, standardized mean difference; IHD, ischemia heart disease; CKD, chronic kidney disease; HMG-CoA, 3-hydroxy-3-methylglutaryl coenzyme A; ACE inhibitors, angiotensin-converting enzyme inhibitors; ARBs, angiotensin II receptor blockers; DPP-4 inhibitors, dipeptidyl peptidase-4 inhibitors; COVID-19, coronavirus disease 2019; BMI, body mass index; HbA1c, glycated hemoglobin A1c; LDL-C, low-density lipoprotein cholesterol; ALT, alanine aminotransferase; TG, triglycerides; CRP, C-reactive protein; BNP, B-type natriuretic peptide. Percentages are rounded to one decimal place. SMD is rounded to two decimal places.


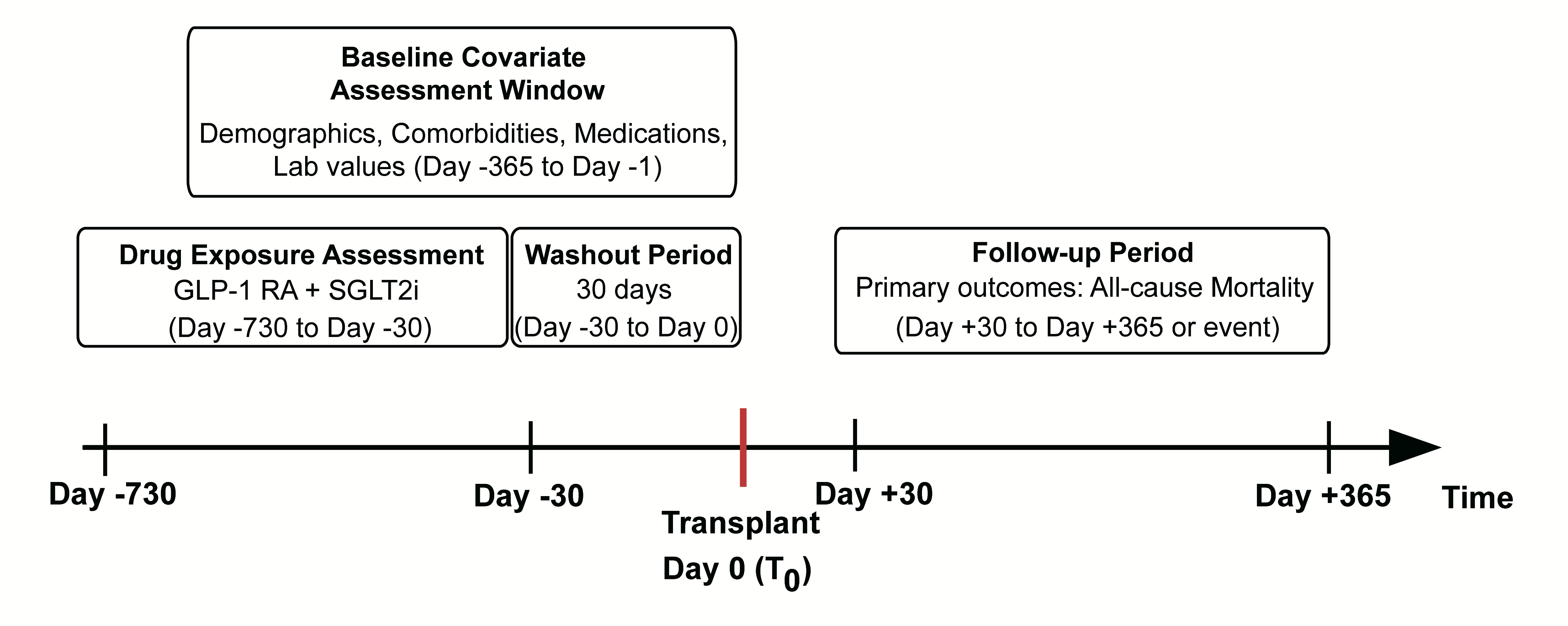


Supplementary **Figure 1. Target trial emulation design for pre-transplant dual metabolic therapy in organ transplant candidates.** The timeline illustrates the systematic approach to evaluate pre-transplant GLP-1 receptor agonist plus SGLT2 inhibitor combination therapy. The baseline covariate assessment window spans from Day -365 to Day -1 relative to transplantation, capturing demographics, comorbidities, concomitant medications, and laboratory parameters during the 12 months preceding surgery. Drug exposure assessment occurs from Day -730 to Day -30, defining the pre-transplant metabolic optimization period where patients receive dual GLP-1 RA plus SGLT2i therapy, monotherapy with either agent, or usual care. A 30-day washout period (Day -30 to Day 0) precedes transplantation to minimize peri-operative confounding and ensure medication effects are attributable to pre-transplant exposure rather than immediate post-surgical management. The index date (T₀) represents the day of organ transplantation. Follow-up commences 1-day post-transplant and continues until the first occurrence of primary outcomes (all-cause mortality), secondary outcomes (kidney transplant complications, rejection, failure, infection-related and adverse events), loss to follow-up, or administrative censoring at Day +365. This design framework emulates the conditions of a randomized controlled trial while leveraging real-world electronic health record data to assess the association between pre-transplant metabolic preparation and post-transplant outcomes in kidney transplant recipients with type 2 diabetes and obesity.





Supplementary **Figure 2. As-treated post-transplant exposure strata (no post-index, 0–3 months, 0–6 months) and 12-month hazards for mortality and graft events comparing dual GLP-1 RA+SGLT2i with GLP-1 RA, SGLT2i, and usual care.**





Supplementary **Figure 3. Infection-related outcomes at 3, 6, and 12 months post-transplantation.** Forest plots show adjusted hazard ratios (95% CI) for infection outcomes comparing dual GLP-1 RA plus SGLT2i therapy with GLP-1 RA monotherapy, SGLT2i monotherapy, and usual care at 3, 6, and 12 months follow-up. Outcomes include cytomegaloviral disease, candidiasis, aspergillosis, mycoses, all-cause sepsis, pneumonia, respiratory failure, and severe sepsis. Green diamonds indicate statistically significant results favoring dual therapy; gray circles represent non-significant findings. NCO were included to assess residual confounding. GLP-1 RA, glucagon-like peptide-1 receptor agonist; NCO, negative control outcome; SGLT2i, sodium-glucose cotransporter-2 inhibitor.

**
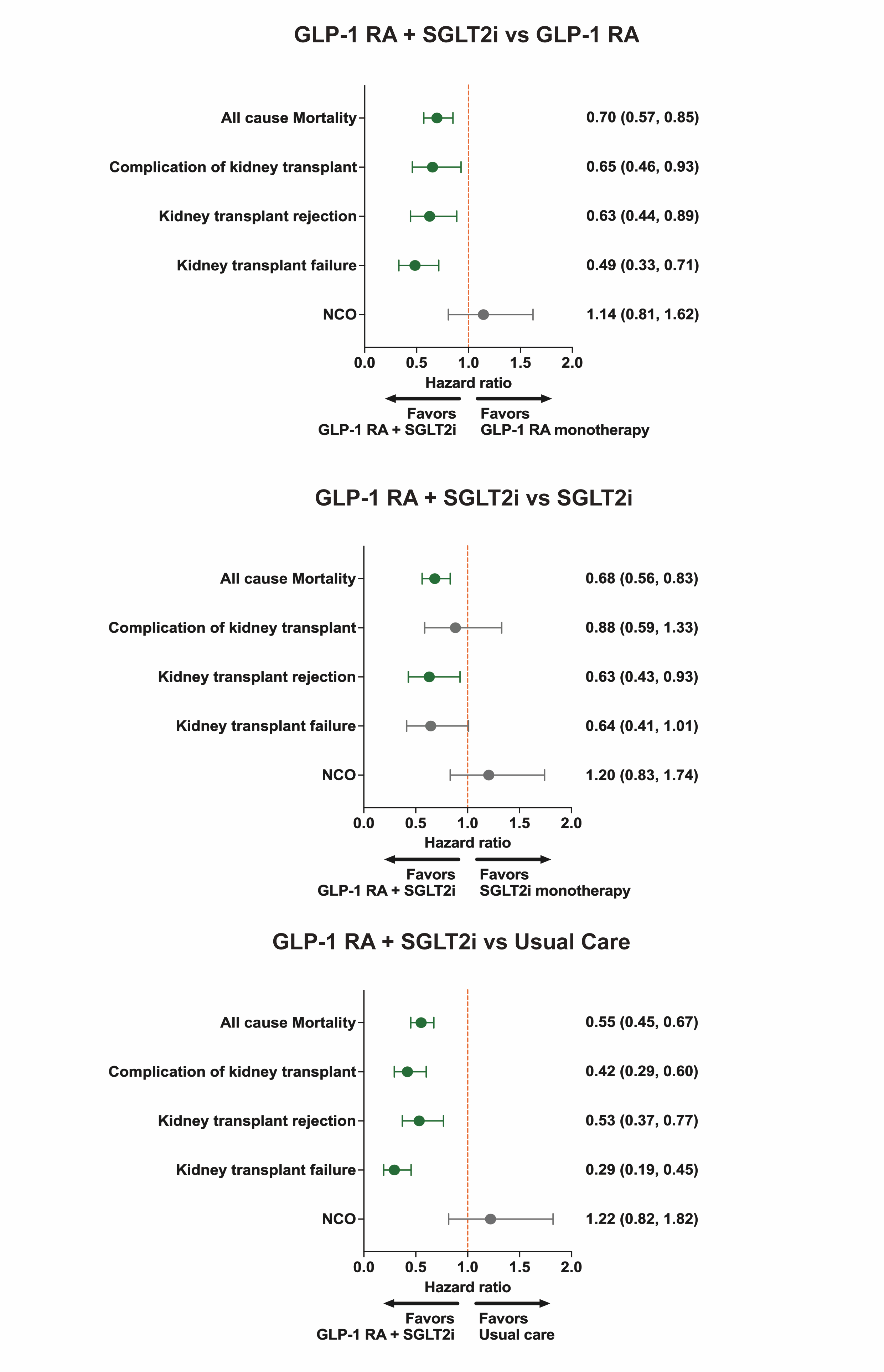
**

**Supplementary Figure 4. Sensitivity analysis restricted to global collaborative network at 12 months.**Forest plots show adjusted hazard ratios (95% CI) for transplant outcomes comparing dual GLP-1 RA plus SGLT2i therapy with GLP-1 RA monotherapy (top), SGLT2i monotherapy (middle), and usual care (bottom) using data from North America, Europe, and Asia-Pacific healthcare organizations. Outcomes include all-cause mortality, transplant complications, rejection, and graft failure at 12 months. Green diamonds indicate statistically significant results favoring dual therapy; gray circles represent non-significant findings. NCO assess residual confounding. GLP-1 RA, glucagon-like peptide-1 receptor agonist; NCO, negative control outcome; SGLT2i, sodium-glucose cotransporter-2 inhibitor.


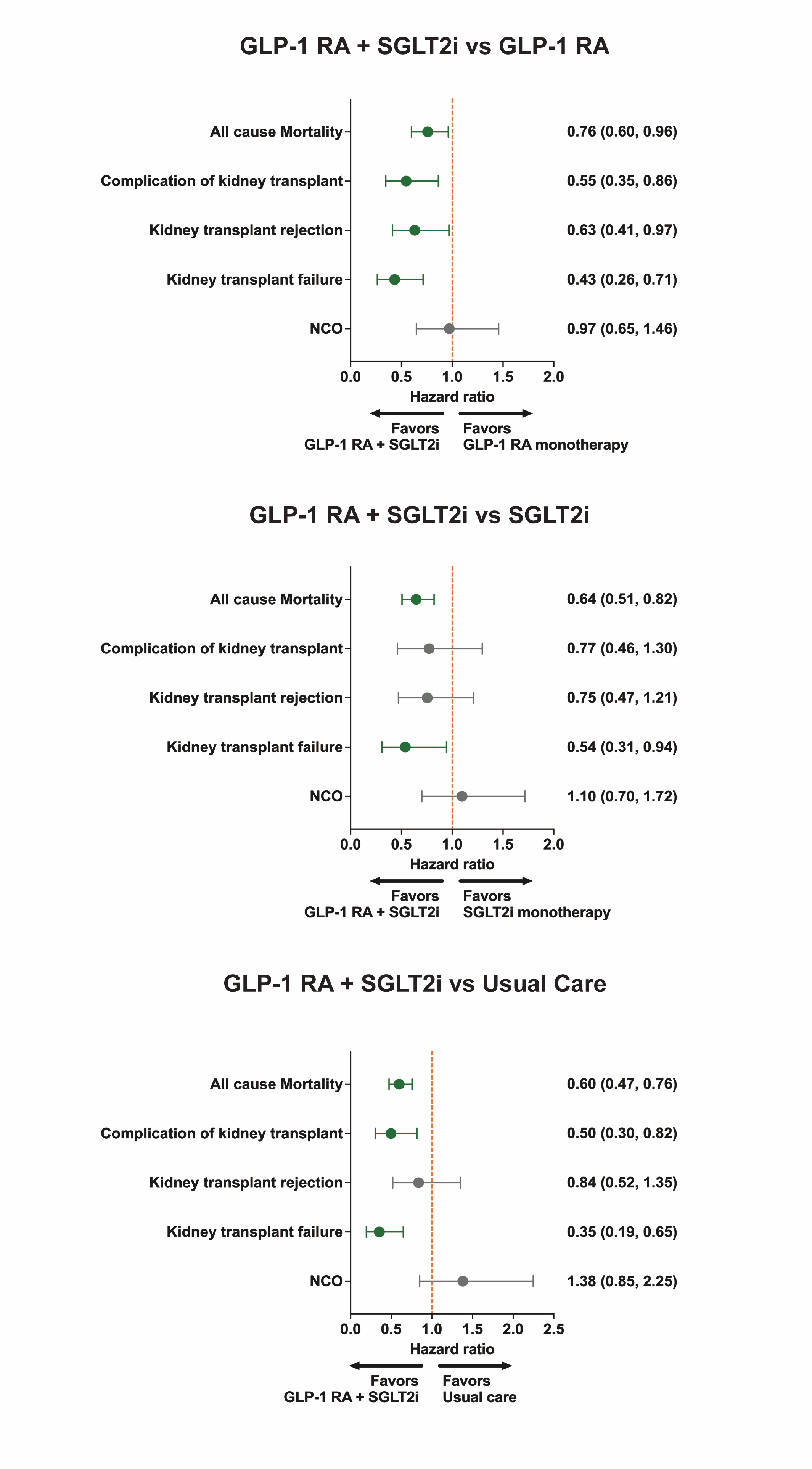


**Supplementary Figure 5. Landmark analysis of transplant outcomes from 3 to 12 months post-transplantation.** Forest plots show adjusted hazard ratios (95% CI) for transplant outcomes comparing dual GLP-1 RA plus SGLT2i therapy with GLP-1 RA monotherapy (top), SGLT2i monotherapy (middle), and usual care (bottom) during the 3**–**12 months follow-up period. This landmark analysis excludes events occurring in the first 6 months post-transplantation to assess sustained treatment effects. Primary outcomes include all-cause mortality, transplant complications, rejection, and graft failure. Green diamonds indicate statistically significant results favoring dual therapy; gray circles represent non-significant findings. NCO assess residual confounding. GLP-1 RA, glucagon-like peptide-1 receptor agonist; NCO, negative control outcome; SGLT2i, sodium-glucose cotransporter-2 inhibitor.


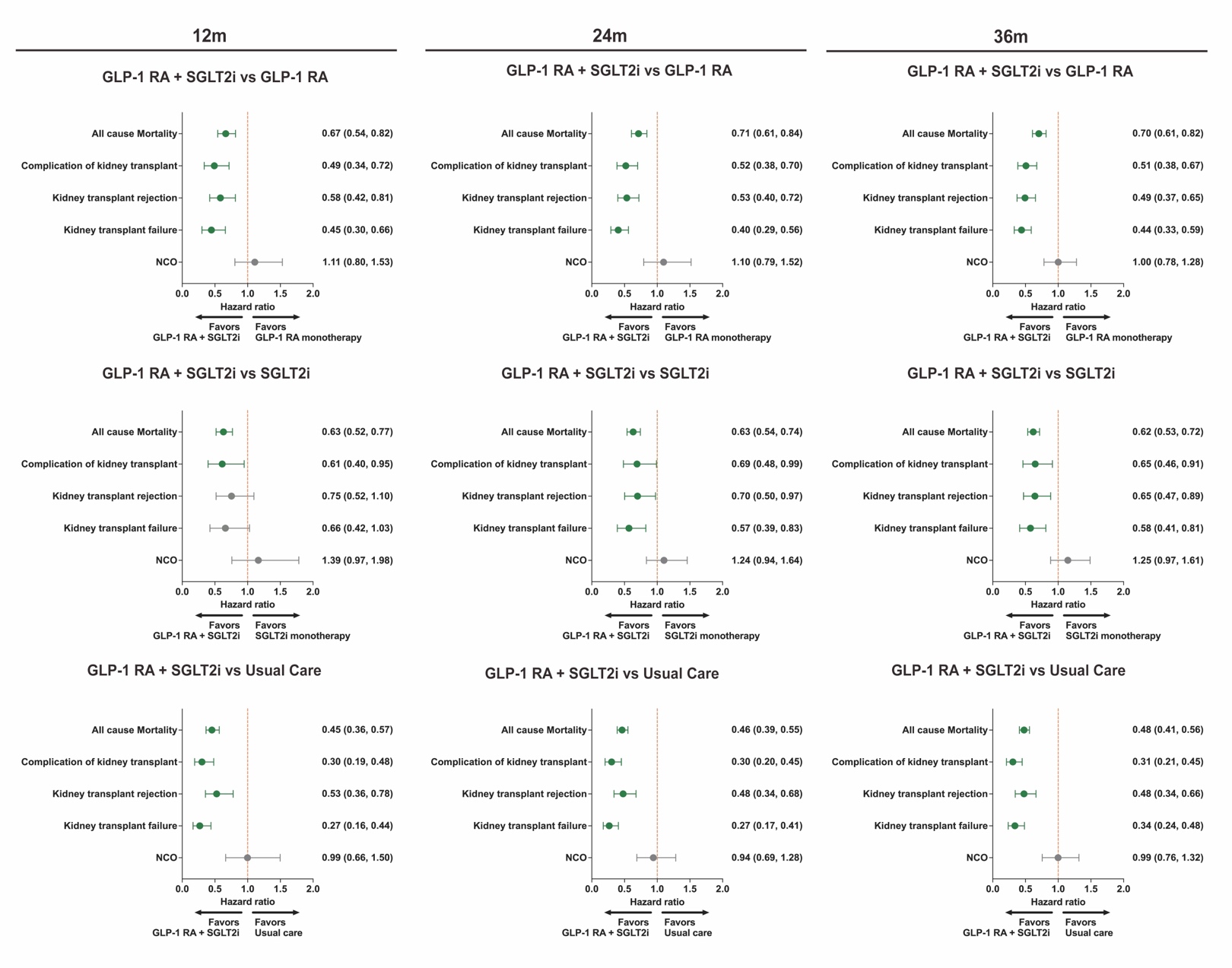


**Supplementary Figure 6. Early-use sensitivity analysis with a 12-month pre-transplant exposure window.** Hazard ratios from 1 to 3 years outcomes comparing pre-transplant dual GLP-1 receptor agonist plus SGLT2 inhibitor therapy with GLP-1 receptor agonist monotherapy, SGLT2 inhibitor monotherapy, and usual care, when exposure is defined only within 12 to 1 months before transplantation. Follow-up for all outcomes starts at the transplant date and continues up to 36 months. Estimates are derived from propensity score–matched Cox models and are reported for all-cause mortality, kidney-transplant failure, kidney-transplant rejection, transplant complications, and the negative-control outcome. HRs are shown with 95% confidence intervals. Abbreviations: GLP-1 RA, glucagon-like peptide-1 receptor agonist; SGLT2i, sodium–glucose cotransporter-2 inhibitor; NCO, negative-control outcome; HR, hazard ratio; CI, confidence interval.


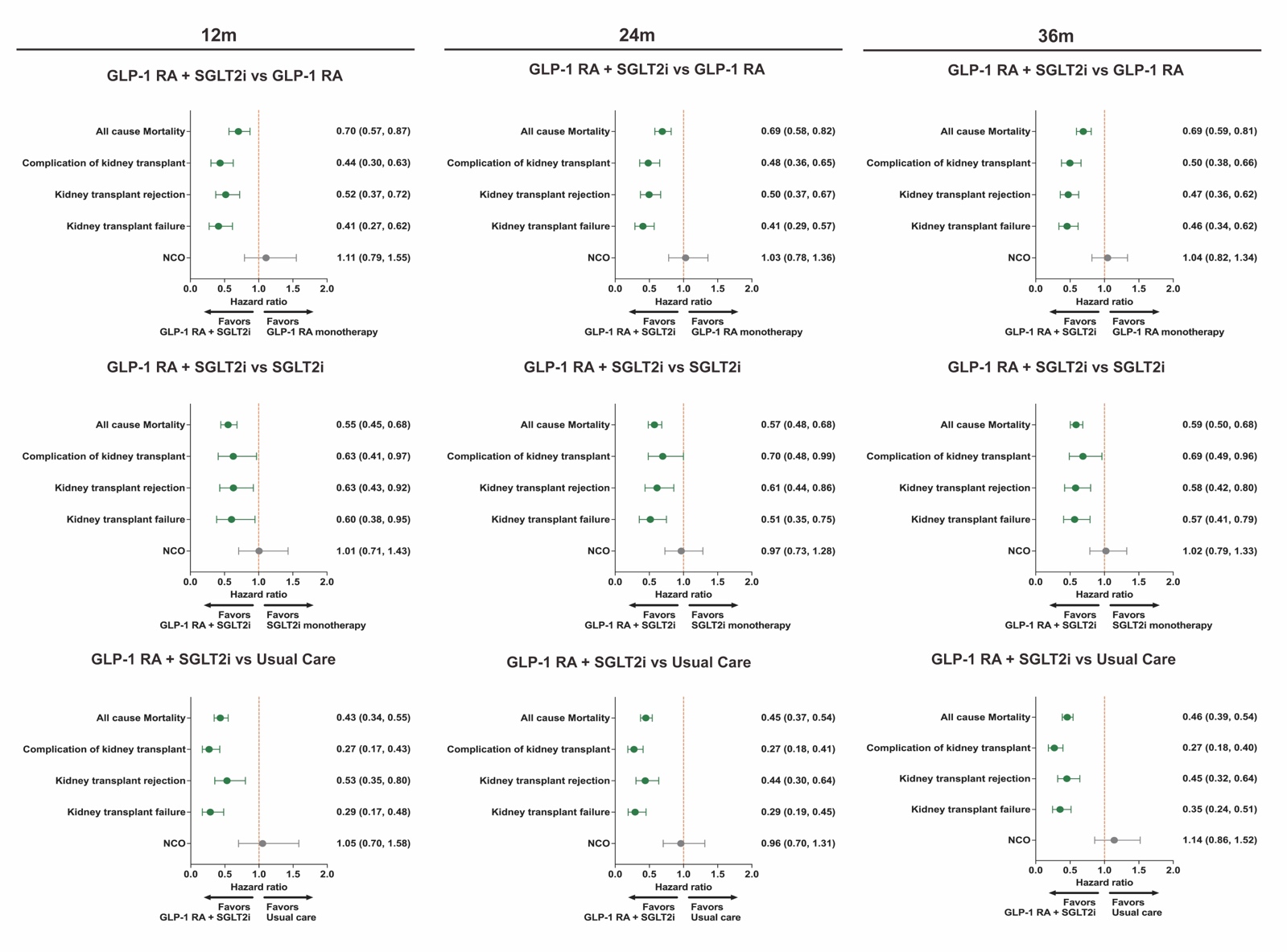


Supplementary Figure 7. Early-use sensitivity analysis with a 6-month pre-transplant exposure window. Hazard ratios from 1 to 3 years outcomes comparing pre-transplant dual GLP-1 RA plus SGLT2i therapy with GLP-1 RA monotherapy, SGLT2i monotherapy, and usual care, when exposure is defined only within 6 to 1 months before transplantation. The transplant date is used as time zero, with follow-up through 36 months. Estimates are obtained from the same propensity score–matched Cox models as in the main analysis and include all-cause mortality, kidney-transplant failure, kidney-transplant rejection, transplant complications, and the negative-control outcome. HRs are presented with 95% confidence intervals. Abbreviations: GLP-1 RA, glucagon-like peptide-1 receptor agonist; SGLT2i, sodium–glucose cotransporter-2 inhibitor; NCO, negative-control outcome; HR, hazard ratio; CI, confidence interval.


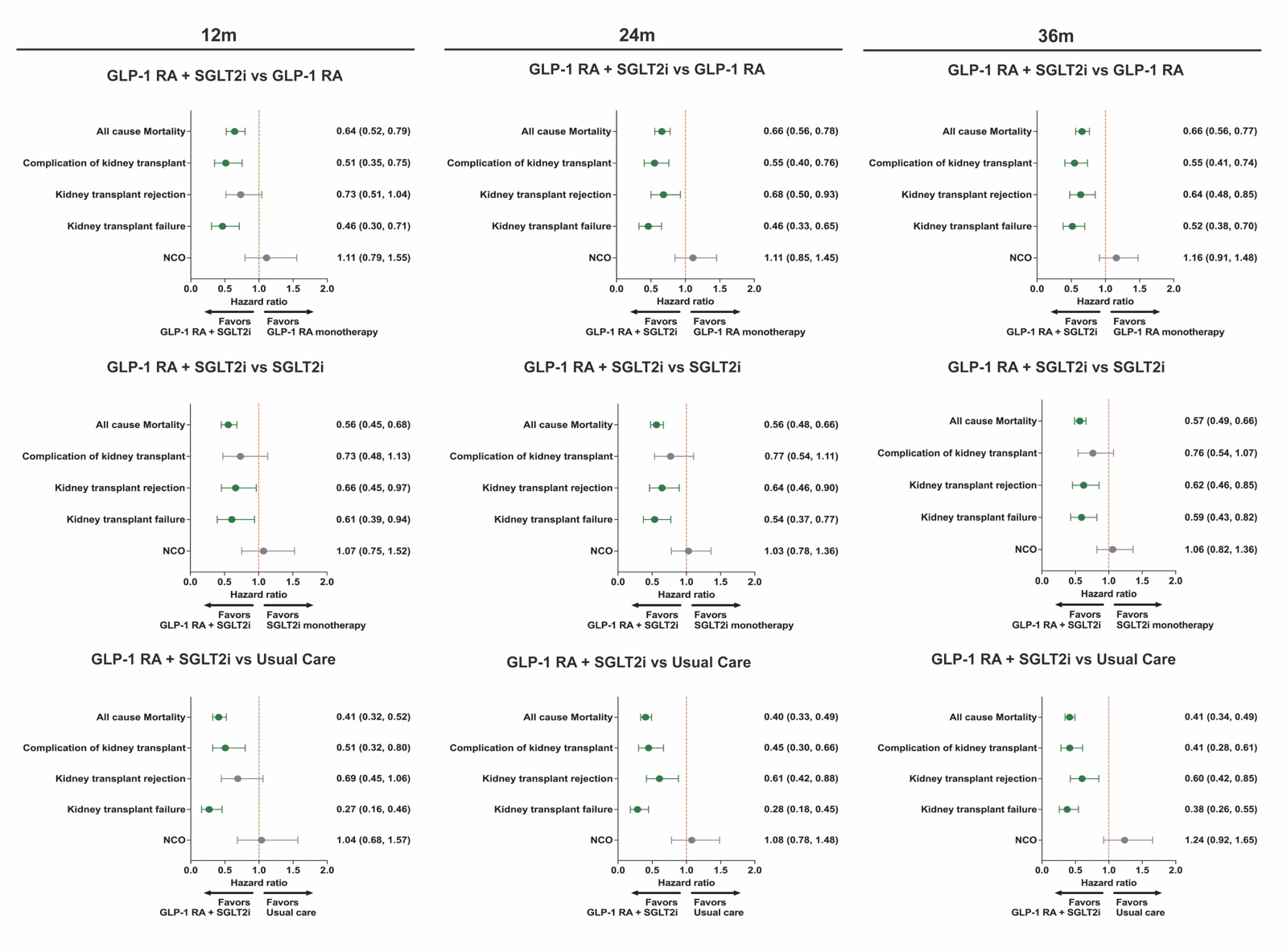


Supplementary Figure 8. Index-at-treatment new-user analysis with a 12-month washout period. Hazard ratios from 1 to 3 years outcomes in the new-user cohort, comparing dual GLP-1 RA plus SGLT2i new users with SGLT2i new users and with never users (usual care). Patients were required to have at least 12 months without GLP-1 RA or SGLT2i prescriptions before the first post-washout prescription in the year before transplantation;• treatment group was assigned at this first prescription, and follow-up for outcomes started at the transplant date and continued up to 36 months. Propensity score–matched Cox models were used to estimate HRs for all-cause mortality, kidney-transplant failure, kidney-transplant rejection, transplant complications, and the negative-control outcome. HRs are shown with 95% confidence intervals. Abbreviations: GLP-1 RA, glucagon-like peptide-1 receptor agonist; SGLT2i, sodium–glucose cotransporter-2 inhibitor; NCO, negative-control outcome; HR, hazard ratio; CI, confidence interval.


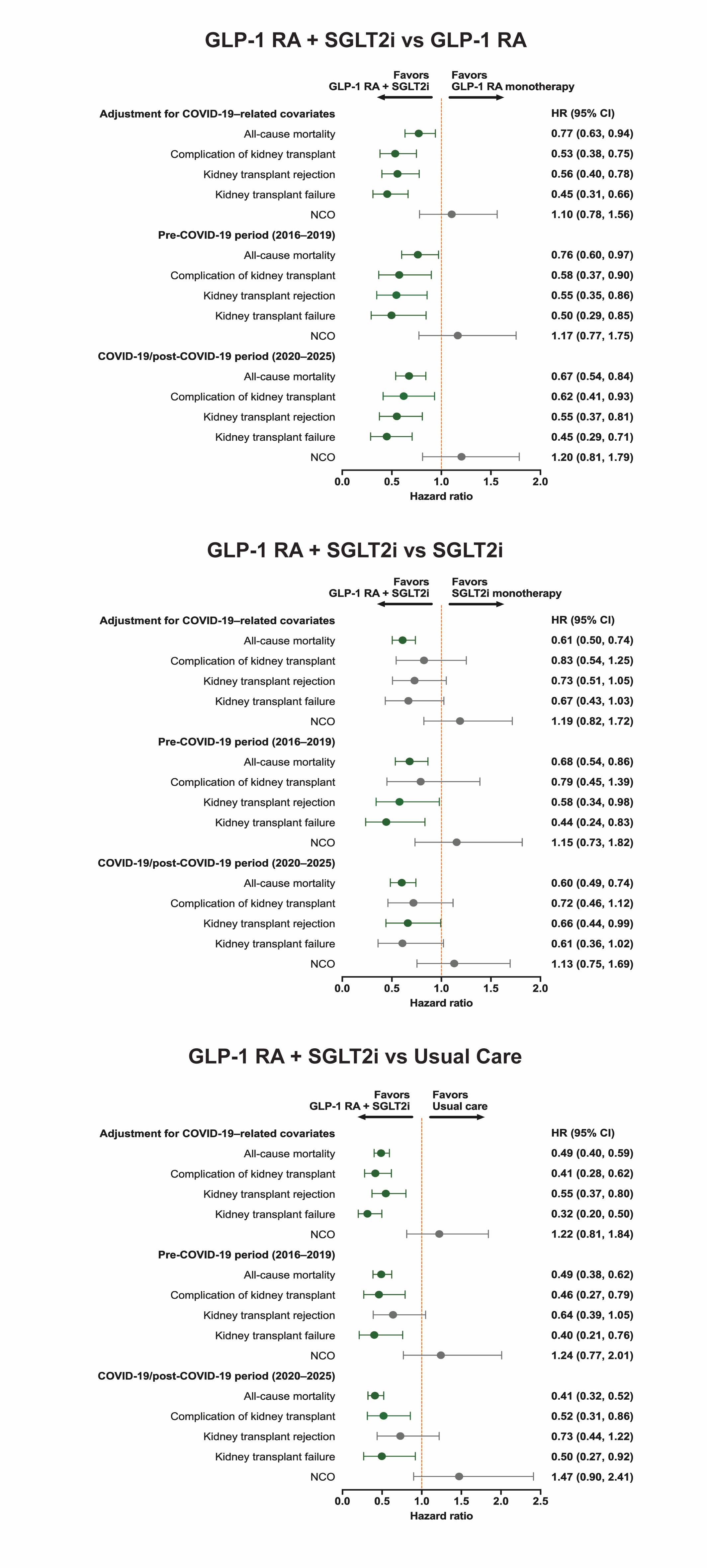


**Supplementary Figure 9. COVID-19–adjusted 12-month intention-to-treat estimates and pre-pandemic sensitivity, by comparator. Forest plots of hazard ratios (HRs) with 95% confidence intervals from Cox models in 1:1 propensity score–matched cohorts comparing pre-transplant dual GLP-1 RA plus SGLT2i with GLP-1 RA monotherapy, SGLT2i monotherapy, and usual care at 12 months. Models add COVID-19 covariates captured in the 12-month baseline: ICD-10 U07.1 (COVID-19 diagnosis), ICD-10 U09.9 (post-COVID condition), vaccination (CVX 213), and SARS-CoV-2 RNA/antibody testing (LOINC-type 9088/9089). The negative-control outcome (skin cancer) is displayed to assess residual bias. A calendar-time sensitivity restricted to 2016–2019 is shown for mortality (panel D) to address pandemic-era confounding. Values <1.0 favor dual therapy. GLP-1 RA, glucagon-like peptide-1 receptor agonist; SGLT2i, sodium–glucose cotransporter-2 inhibitor; HR, hazard ratio; CI, confidence interval.**


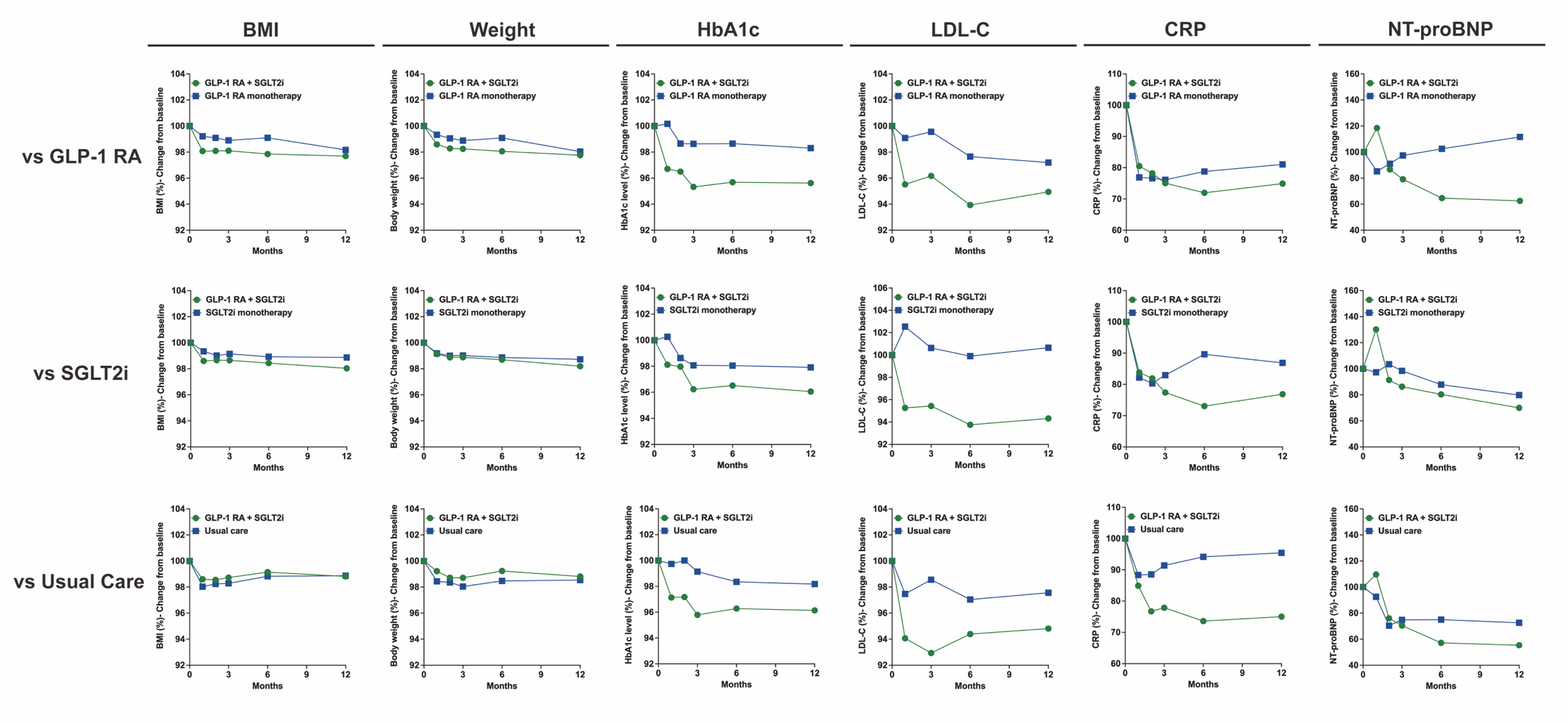


**Supplementary Figure 10. Longitudinal post-transplant biomarkers through 360 days in matched cohorts (intention-to-treat). Line plots show group means (points) for HbA1c (%), BMI (kg/m²), weight (lb), LDL-C (mg/dL), CRP (mg/L), and NT-proBNP (pg/mL) at 0, 90, 180, and 360 days after transplantation in the 1:1 matched cohorts. Contrasts display mean differences (dual therapy – comparator; green color) at each time point; *p*-values are from two-sided independent-samples t-tests and are descriptive (no multiplicity adjustment). Analyses followed intention-to-treat cohort assignment and used observed values without imputation. HbA1c, glycated hemoglobin A1c; BMI, body mass index; LDL-C, low-density lipoprotein cholesterol; CRP, C-reactive protein; NT-proBNP, N-terminal pro–B-type natriuretic peptide; GLP-1 RA, glucagon-like peptide-1 receptor agonist; SGLT2i, sodium–glucose cotransporter-2 inhibitor.**
